# EML4-ALK Variant 3 Promotes Mitotic Errors and Spindle Assembly Checkpoint Deficiency Leading to Increased Microtubule Poison Sensitivity

**DOI:** 10.1158/1541-7786.MCR-21-1010

**Published:** 2022-02-25

**Authors:** Kellie Lucken, Laura O'Regan, Jene Choi, Josephina Sampson, Sarah L. Pashley, Richard Bayliss, Sam Khan, Andrew M. Fry

**Affiliations:** 1 Department of Molecular and Cell Biology, University of Leicester, Leicester, United Kingdom.; 2 Department of Pathology, Asan Medical Center, University of Ulsan College of Medicine, Seoul, South Korea.; 3 School of Molecular and Cellular Biology, Astbury Centre for Structural Molecular Biology, Faculty of Biological Sciences, University of Leeds, Leeds, United Kingdom.; 4 Leicester Cancer Research Centre, Department of Genetics and Genome Biology, University of Leicester, Robert Kilpatrick Clinical Sciences Building, Leicester, United Kingdom.

## Abstract

EML4-ALK is an oncogenic fusion protein present in approximately 5% of non–small cell lung cancers (NSCLC). Alternative breakpoints in the gene encoding EML4 result in distinct variants that are linked to markedly different patient outcomes. Patients with EML4-ALK variant 3 (V3) respond poorly to ALK inhibitors and have lower survival rates compared with patients with other common variants, such as V1. Here, we use isogenic Beas-2B bronchial epithelial cell lines expressing EML4-ALK V1 or V3, as well as ALK-positive NSCLC patient cells that express V1 (H3122 cells) or V3 (H2228 cells), to show that EML4-ALK V3 but not V1 leads to hyperstabilized K-fibers in mitosis, as well as errors in chromosome congression and segregation. This is consistent with our observation that EML4-ALK V3 but not V1 localizes to spindle microtubules and that wild-type EML4 is a microtubule stabilizing protein. In addition, cells expressing EML4-ALK V3 exhibit loss of spindle assembly checkpoint control that is at least in part dependent on ALK catalytic activity. Finally, we demonstrate that cells expressing EML4-ALK V3 have increased sensitivity to microtubule poisons that interfere with mitotic spindle assembly, whereas combination treatment with paclitaxel and clinically approved ALK inhibitors leads to a synergistic response in terms of reduced survival of H2228 cells.

**Implications:**

This study suggests that combining the microtubule poison, paclitaxel, with targeted ALK inhibitors may provide an effective new treatment option for patients with NSCLC with tumors that express the EML4-ALK V3 oncogenic fusion.

## Introduction

Lung cancer is the most prevalent cancer worldwide and has one of the lowest survival rates (1). Although significant advances have been made in treatment for many other cancers, the 10-year survival rate of lung cancers remains stubbornly low at ∼10% (1, 2). On the basis of histologic classification, the majority are non–small cell lung cancers (NSCLC) and these can be further subdivided into adenocarcinomas, squamous cell carcinomas, and large cell carcinomas. Molecular profiling of lung adenocarcinomas revealed that ∼5% are driven by the oncogenic fusion protein, EML4-ALK (3), which arises from a chromosomal inversion event on the short arm of chromosome 2. This results in an in-frame fusion of the N-terminus of echinoderm microtubule-associated protein (EMAP)-like 4 (EML4) protein with the C-terminal catalytic domain of anaplastic lymphoma kinase, ALK (3). Targeted ALK inhibitors, including crizotinib, ceritinib, alectinib, and lorlatinib, have proven highly successful for treatment of patients with ALK-positive NSCLC and are the current standard of care (4).

The wild-type EML4 protein has a coiled-coil trimerization domain (TD) at the N-terminus, followed by an unstructured basic linker and a C-terminal “TAPE” domain comprised of a pair of seven-bladed β-propellers formed from multiple WD repeats (5–7). EML4 is a microtubule-associated protein that binds microtubules via its N-terminal TD and basic linker, whereas the TAPE domain binds to soluble α/β-tubulin heterodimers. EML4 has been reported to promote microtubule stabilization although the mechanism for this remains unclear (5, 8). Interestingly, although EML4 decorates the outer surface of the microtubule lattice in interphase, phosphorylation of two highly conserved residues (S144 and S146) in the basic linker results in its dissociation from microtubules in mitosis (8, 9). This is necessary for spindle assembly as expression of an S144/146A mutant leads to inappropriate association with the mitotic spindle, as well as misaligned chromosomes in metaphase and delayed anaphase onset (8). These two sites can be phosphorylated by the mitotic kinases, NEK6 and NEK7, *in vitro* and siRNA depletion of NEK6 and NEK7, or their upstream activator, NEK9, also causes EML4 to localize to spindle microtubules (8). Interestingly, although displacement of EML4 from microtubules appears important for spindle function, it nevertheless remains essential for mitosis as EML4 depletion promotes chromosome misalignment, activation of the spindle assembly checkpoint (SAC) and delayed anaphase onset (8, 9).

Since discovery of the EML4-ALK fusion, as many as 15 distinct variants have been identified due to variation in the breakpoint of the *EML4* gene (3, 10–12). However, all variants include the TD of EML4 allowing trimerization of EML4-ALK and constitutive activation of the ALK catalytic domain, presumably through auto-phosphorylation (3, 5, 13). This leads to signaling through various oncogenic pathways, including PI3K/AKT, JAK/STAT, and RAS/MAPK, that promote tumor progression and survival (14, 15). The most prevalent EML4-ALK variants are variant 1 (V1, 33%) and variant 3 (V3, 30%; ref. 16). V3 is a relatively short variant encoding none of the TAPE domain of EML4, whereas V1 is much larger and encodes a large portion of this domain (17, 18). Translation of an incomplete TAPE domain makes V1 an unstable protein that requires the molecular chaperone HSP90 for its expression (17, 18). Importantly, response of patients to ALK inhibitors also varies and is dependent on the specific EML4-ALK variant expressed with patients with V1 displaying higher progression-free survival (PFS) than patients with V3 (17, 19).

Surprisingly, although each variant contains the N-terminal TD and at least some of the basic linker of EML4 essential for microtubule localization, only V3 (of those variants tested) localizes to interphase microtubules with the same propensity as wild-type EML4 (5). This inability of the longer variants, such as V1, to associate with microtubules could be due to misfolding or interaction with HSP90, either of which could mask the microtubule-binding site (18). Interestingly, expression of recombinant V3 in U2OS cells or endogenous V3 in the ALK-positive NSCLC patient cell line, H2228, leads to altered cell morphology with elongated cytoplasmic protrusions as well as enhanced cell migration, potentially as a result of increased microtubule stabilization (20). Moreover, these phenotypes are dependent on NEK7 and NEK9, which are recruited to interphase microtubules by the V3 protein (20). EML4-ALK variants also form phase-separated liquid droplets in the cytoplasm where they recruit downstream ALK effector proteins (21–23). Both V1 and V3 droplets have been detected, albeit with V3 droplets being more dynamic than V1 droplets. Interestingly, treatment with certain targeted ALK inhibitors causes these droplets to disperse suggesting that their formation is dependent on catalytic activity (22). This has led to the hypothesis that ALK signaling in NSCLC cells preferentially occurs from within the liquid droplets, whereas the V3 protein detected on microtubules may be less active (20, 22).

Here, we present evidence that the EML4-ALK V3 protein localizes not only to interphase microtubules but also to the mitotic spindle, and that its expression increases the frequency of mitotic errors. It also interferes with SAC function raising the prospect that EML4-ALK V3 may accelerate genomic instability. Finally, we show that when H2228 cells expressing V3 are treated with a combination of the microtubule stabilizing drug, paclitaxel, and targeted ALK inhibitors they exhibit a synergistic response raising the prospect of a more effective approach to treating patients with NSCLC with EML4-ALK V3.

## Materials and Methods

### Cell culture and transfection

Parental and isogenic Beas-2B bronchial epithelial cells engineered to express either HA-EML4-ALK V1 or V3 upon induction with doxycycline have been described previously (20). H3122 and H2228 cells were obtained from ATCC within the last 10 years. Beas-2B, H3122, and H2228 cell lines were cultured in RPMI media (Invitrogen) supplemented with 10% FBS and penicillin–streptomycin (PS, 100 and 100 U/mL, respectively) at 37°C, 5% CO_2_. Beas-2B HA-EML4-ALK V1 and V3 cells were cultured in RPMI media with FBS and PS, as well as 0.25 μg/mL puromycin and 100 μg/mL G418. Cells were stored in liquid nitrogen and cultured for a maximum of 2 months and checked for mycoplasma infection by in-house PCR every 2 months. To induce the expression of HA-EML4-ALK V1 or V3, cells were treated with 1 μg/mL doxycycline for 48 hours. Once cells reached appropriate confluency, they were passaged by removing media and washing in PBS (137 mmol/L NaCl, 2.7 mmol/L KCl, 8.1 mmol/L Na_2_HPO_4_, 1.5 mmol/L KH_2_PO_4_, pH 7.4). Cells were detached using 1× PBS containing 0.5 mmol/L EDTA. YFP-EML4-ALK V3 constructs including the D1270N kinase-dead (KD) plasmid were constructed as described previously (20). For transfection, cells were seeded at an appropriate density such that they were 60% confluent on the day of transfection. Plasmid DNA and Fugene transfection reagent were diluted at a 1:3 ratio in OptiMEM reduced serum media (Invitrogen) according to manufacturer's instructions. Transfection mixture was added to cells in a dropwise manner and cells incubated at 37°C 5% CO_2_ for 24 hours before being processed for Western blot, immunofluorescence microscopy, flow cytometry, or live cell imaging analysis. Cell lines used in this study had been authenticated at point of acquisition or purchase, were not passaged in culture for longer than two months, and are available upon request.

### Drug treatments

Cells were treated with 0.5 µmol/L nocodazole (Sigma, M1404) or 5 µmol/L paclitaxel (Sigma, T7191), diluted in fresh media for the indicated time periods. ALK inhibitors were obtained from Selleck and reconstituted in DMSO: alectinib (S2762), ceritinib (S7083), and crizotinib (S1068). TAE-684 was obtained from MedChemExpress and reconstituted in DMSO.

### siRNA depletion

Cells were seeded in a 6-well dish such that they reached 30% to 40% confluency on the day of transfection in OptiMEM reduced serum media (Invitrogen) with 10% FBS and no antibiotics. After 24 hours, 100 nmol/L siRNA oligonucleotides and oligofectamine (Invitrogen) were combined in OptiMEM according to manufacturer's instructions. Cells were washed in OptiMEM without FBS, and the mixture added to cells in a dropwise manner and incubated at 37°C 5% CO_2_ for 4 hours. OptiMEM containing 30% FBS was then added and incubated a further 72 hours before processing for Western blotting or immunofluorescence microscopy.

### Preparation of cell extracts, SDS-PAGE, and Western blotting

Cells were lysed in RIPA lysis buffer [50 mmol/L Tris-HCl pH 8, 150 mmol/L NaCl, 1% v/v Nonidet P-40, 0.1% w/v SDS, 0.5% w/v sodium deoxycholate, 5 mmol/L NaF, 5 mmol/L β-glycerophosphate, 30 µg/mL RNase, 30 µg/mL DNase I, 1× Protease Inhibitor Cocktail (Melford Laboratories), 1 mmol/L PMSF] followed by analysis by SDS-PAGE and Western blotting. Primary antibodies used were against: HA (1:1,000, Sigma H3663), α-tubulin (1:5,000, mouse clone B512, Sigma T5168), α-tubulin (1:5,000, rabbit antibody, Abcam ab15246), ALK (1:1,000, Cell Signaling Technology 3633), ERK (1:2,000, Cell Signaling Technology 9102), and pERK (1:1,000, Cell Signaling Technology 9101). Secondary antibodies used were horseradish peroxidase (HRP)-labeled IgGs against rabbit (1:2,000; Sigma A6154) and mouse (1:2,000, Bethyl Laboratories A90–116P) antibodies. Protein bands were visualized by enhanced chemiluminescence (ECL) according to manufacturer's instructions (Pierce).

### Fixed and live-cell microscopy

Cells grown on acid-etched coverslips were fixed with ice-cold methanol for 30 minutes. Cells were permeabilized and blocked using 0.2% TritonX-100 PBS with 3% BSA in PBS. Primary antibodies used were against GFP (1:1,000, Abcam, ab6556), HA (1:500, Sigma, H3663), α-tubulin (1:2,000, mouse clone B512, Catalog No. T5168, Sigma), α-tubulin (1:2,000, rabbit antibody, Abcam, ab15246), ALK (1:1,000 WB, Cell Signaling Technology, 3633), BubR1 (1:500, BD Biosciences, Catalog No. 612503), CENP-A (1:500, Abcam, ab13939), γ-tubulin (1:1,000, mouse antibody, Sigma, T6557), γ-tubulin (1:1,000, rabbit antibody, Abcam, ab11321), and centrin-2 (1:500, clone N17 Santa Cruz Biotechnology, sc7396). Secondary antibodies were anti-mouse or anti-rabbit IgGs conjugated with Alexa Fluor-488 or Alexa Fluor-594, and DNA was stained with Hoechst 33258. Staining of cells with ALK antibodies was as previously described with blocking in PBS containing 1% BSA and 0.1% Triton-X-100 (5). Images were captured using a VisiTech Infinity 3 confocal microscope fitted with a Hamamatsu C11440 -22CU Flash 4.0 V2 sCMOS camera and a Plan Apo VC 60x (NA 1.4) or Plan Apo 100 x objective (NA 1.47) at 0.25 μm z-slices and analyzed using FIJI. SRRF reconstructions were generated using the nano-J SRRF plugin in FIJI. Live cell imaging of mitotic progression was performed on cells cultured in 6-well dishes and imaged using a LiveCyte 2 (PhaseFocus) microscope at 37°C 5% CO_2_ for 24 hours with images captured every 5 minutes. Mitotic duration was calculated for 50 cells per condition and scored from when the cells began to round up upon mitotic entry until the time cells began to divide and there was a clear distinction between the two daughter cells undergoing cytokinesis.

### Flow cytometry

For cell-cycle analysis by flow cytometry, cells were harvested in 1× trypsin (Invitrogen) and centrifuged, then washed in PBS, and fixed in ice cold 70% ethanol overnight. Cells were washed again in 1× PBS before staining with 1× PBS containing 200 µg/mL RNAse A and 20 µg/mL propidium iodide (PI; Abcam) for 4 hours in the dark at 4°C. Stained cells were analyzed by flow cytometry on a Beckman Coulter CytoFlex flow cytometer to determine cell-cycle distribution. For cell-cycle analyses of YFP positive cells, cells were gated based on untransfected control cells and only YFP-positive cells analyzed for cell-cycle distribution using PI. DNA peaks resulting from PI staining were gated manually based on the *n* and 2*n* populations of control untreated samples and kept constant across repeat experiments. To determine the percentage of cells in apoptosis, cells were grown in 6 cm dishes. All cells were harvested and washed in fresh prewarmed complete growth media, and allowed to recover at 37°C 5% CO_2_ for 30 minutes. Cells were then pelleted by centrifugation and washed in PBS. Cells were stained using the Abcam Annexin-V FITC Apoptosis Detection Kit (Ab14085) according to manufacturer's instructions and incubated for 15 minutes at room temperature in the dark. Stained cells were analyzed by flow cytometry on a CytoFlex flow cytometer (Beckman Coulter).

### Clonogenic assays

Cells were seeded in 6-well dishes and drugs described added the following day for 24 hours. Two hundred live cells were then seeded into 6-well dishes in triplicate and left to grow for 10 to 14 days or until colonies of 50 or more cells were visible. Wells were washed with *dd*H_2_O and fixed in 100% methanol for 2 minutes. Colonies were then stained with 0.5% crystal violet (Sigma) for 1 minute and left to air dry before being counted. To calculate the plating efficiency (PE) of each treatment, the following calculation was used:




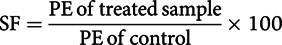




Using the PE for each treatment, the surviving fraction (SF) was calculated using the following equation:




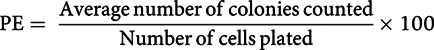




### Alamar blue cell viability assay

A total of 4,000 cells were seeded into wells of a 96-well plate in triplicate and allowed to adhere for 24 hours. One hundred microliters of the drugs described were diluted in fresh media to the concentration required, added to wells in triplicate, and incubated for 48 hours. Growth media was replaced with 100 µL fresh media with 10% Alamar Blue (Invitrogen) and the plate incubated for 4 hours. Fluorescence was quantified using a HidexSense microplate reader (Hidex) with fluorescence measured at 488 nm. Cell viability was normalized relative to DMSO-treated samples.

### Statistical analysis

Statistical tests on three independent repeats of data collected were conducted using GraphPad Prism version 9.1. Error bars depict the SD of the mean as calculated using GraphPad. For experiments where multiple cells were measured per repeat, statistical significance was calculated on the basis of the mean of each experiment such that *n* = 3 overall. One tailed unpaired Student *T* test was used to determine the statistical difference between the means of two groups and a one-way ANOVA was used for instances where there were more than two groups. *P* values were calculated and considered statistically significant when lower than 0.05 (*, *P* < 0.05; **, *P* < 0.01; ***, *P* < 0.001; ****, *P* < 0.0001; ns, nonsignificant). To determine the IC_50_ of drugs used in dose–response calculations, a nonlinear regression line was calculated using GraphPad Prism from 100% to 0%. To assess whether combinations of ALK TKIs and paclitaxel were synergistic, the Chou–Talalay method in CompuSyn (24, 25).

## Results

### Cells expressing EML4-ALK V3 have increased microtubule stability in mitosis

**Figure 1.**
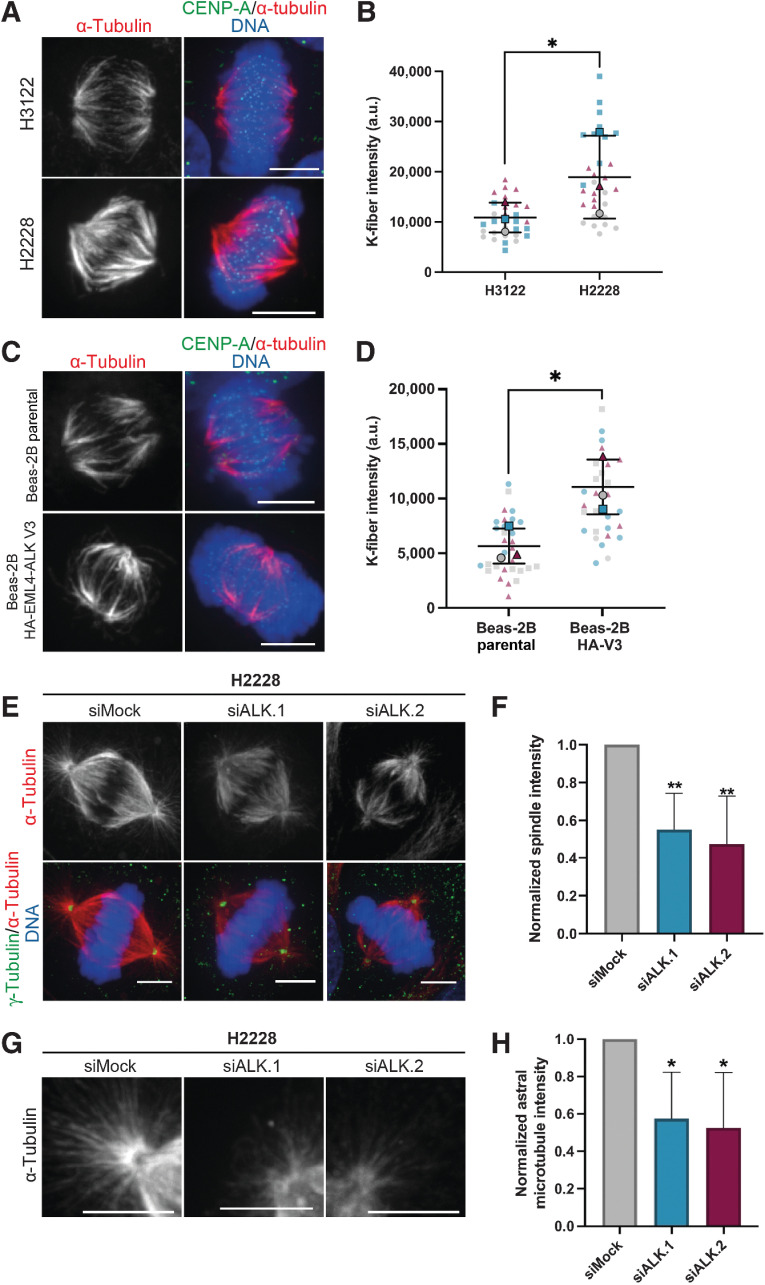
Expression of EML4-ALK V3 increases microtubule stability in mitosis. **A,** H3122 and H2228 cells were incubated on ice for 15 minutes before being fixed and stained with α-tubulin (red) and CENP-A (green) antibodies. DNA was stained with Hoechst 33258 (blue). Scale bars, 5 μm. **B,** Fluorescence intensity (a.u., arbitrary units) of K-fibers measured across half-spindles from cells shown in **A** was quantified using ImageJ. *, *P* < 0.05 compared with H3122 by unpaired *T* test. **C,** Beas-2B parental and HA-EML4-ALK V3 cells were induced with doxycycline for 48 hours and incubated on ice for 15 minutes before being stained with α-tubulin (red) and CENP-A (green) antibodies. DNA was stained with Hoechst 33258 (blue). Scale bars, 5 μm. **D,** Fluorescence intensity of K-fibers from cells shown in **C** was measured as in **B**. *, *P* < 0.05 compared with parental by unpaired *T* test. The three different shapes and colours in the dot plots in **B** and **D** indicate data from three independent experiments, with large shapes referring to means and small shapes the individual data points. **E,** H2228 cells were either mock-depleted (siMock) or depleted of EML4-ALK using siALK.1 or siALK.2 and stained with α-tubulin (red) and γ-tubulin (green) antibodies. DNA was stained with Hoechst 33258 (blue). Scale bars, 5 μm. **F,** Spindle intensity measured across half-spindles from cells shown in **E** was quantified using ImageJ and normalized to the intensity observed in siMock treated cells. **, *P* < 0.01 compared with siMock by one-way ANOVA. **G,** H2228 cells were depleted as in **E** and stained with α-tubulin antibodies. **H,** Astral microtubule intensity from cells shown in **G** was quantified as in **F**. **, *P* < 0.01 compared with siMock by one-way ANOVA. Data are means + SD for three independent experiments. Scale bars, 5 μm.

Overexpression of wild-type EML4 in cells promotes interphase microtubule stabilization as measured by decreased sensitivity to the depolymerizing agent, nocodazole, and reduced microtubule growth rate (8, 26). Furthermore, expression of EML4-ALK V3 promotes interphase microtubule stability as measured by increased levels of acetylated tubulin and reduced depolymerization of the microtubule network upon nocodazole treatment (20). We therefore investigated whether EML4-ALK V3 expression may also alter microtubule stability in mitosis. For this purpose, NSCLC patient-derived cells that express either EML4-ALK V1 (H3122) or V3 (H2228) were incubated on ice for 15 minutes. This treatment leads to depolymerization of most microtubules but leaves spindle microtubules that are attached to kinetochores (K-fibers) in mitotic cells intact. The fluorescence intensity of K-fibers was analyzed by immunofluorescence microscopy with α-tubulin antibodies whereas centromeres were stained with CENP-A antibodies. Strikingly, the intensity of cold-stable K-fibers was significantly higher in H2228 (V3) mitotic cells compared with H3122 (V1) mitotic cells (Fig. 1A and B). To test whether this was a direct result of expression of EML4-ALK V3, an isogenic model using Beas-2B bronchial epithelial cells with doxycycline-inducible expression of HA-EML4-ALK proteins was employed (Supplementary Figs. S1A and S1B). Treatment with doxycycline led to a significant increase in cold-stable K-fiber intensity in mitotic cells expressing HA-EML4-ALK V3 compared with parental Beas-2B cells (Fig. 1C and D). Depletion of EML4-ALK V3 from H2228 (V3) cells using two different siRNA oligonucleotides directed against the ALK kinase domain also led to significantly decreased intensity of spindle and astral microtubules compared with mock-depleted cells (Fig. 1E–H; Supplementary Figs. S1C and S1D). Taken together, these data demonstrate that EML4-ALK V3 leads to increased stabilization of microtubules in mitosis.

### Expression of EML4-ALK V3 leads to increased mitotic errors

**Figure 2.**
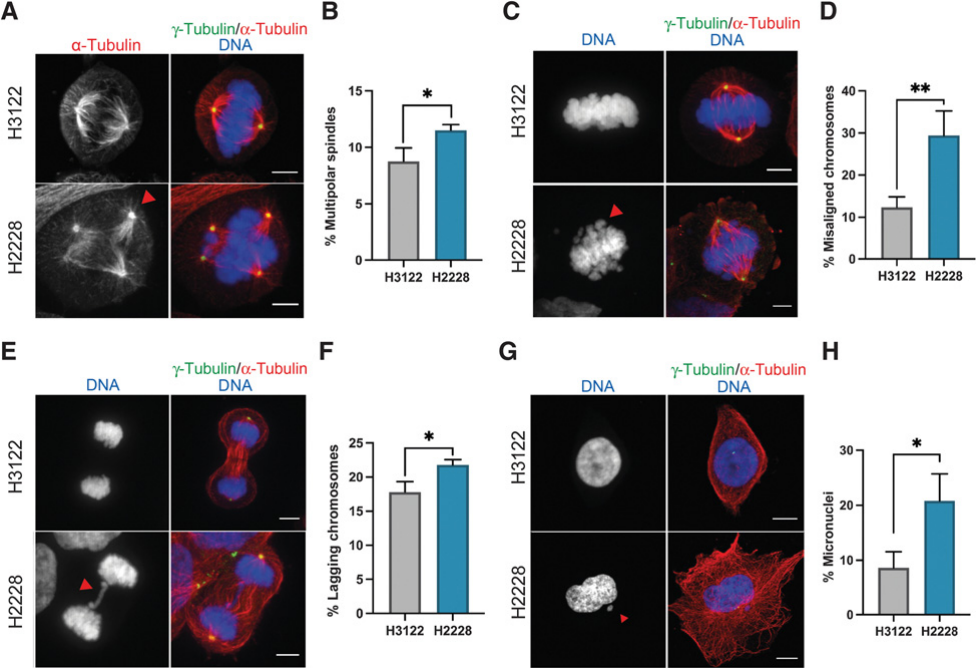
H2228 (V3) cells have increased mitotic errors compared with H3122 (V1) cells. **A, C, E** and **G,** H3122 and H2228 cells were stained with α-tubulin (red) and γ-tubulin (green) antibodies. DNA was stained with Hoechst 33258 (blue). Scale bars in metaphase, 5 μm, and in interphase, 10 μm. **B,** Box plot showing the proportion of cells with multipolar spindles quantified from 100 mitotic cells in **A**. *, *P* < 0.05 compared with H3122 by unpaired *T* test. **D,** Box plot showing the proportion of cells with misaligned chromosomes quantified from 100 bipolar metaphase cells in **C**. **, *P* < 0.01 compared with H3122 by unpaired *t* test. **F,** Box plot showing the proportion of cells with lagging chromosomes quantified from 100 anaphase cells in **E**. *, *P* < 0.05 compared with H3122 by unpaired *t* test. **H,** Box plot showing the proportion of cells with micronuclei quantified from 100 interphase cells in **G**. *, *P* < 0.05 compared with H3122 by unpaired *t* test. Data are means +SD for three independent experiments.

Genome instability is a classic hallmark of cancer cells and contributes to increased malignant properties and tumor evolution (27). As EML4-ALK V3 expressing cells had increased microtubule stabilization in mitosis, we wished to know whether this might lead to mitotic errors that could promote genome instability. Chromosome and spindle organization were therefore examined in H3122 and H2228 mitotic cells by immunofluorescence microscopy based on staining of DNA, α-tubulin and γ-tubulin. Quantitative analysis revealed that H2228 (V3) cells had significantly higher frequencies of both multipolar spindles and misaligned chromosomes (12% and 28%, respectively) than H3122 (V1) cells (8% and 12%, respectively) in metaphase (Fig. 2A–D). Delays in chromosome congression can lead to segregation errors in anaphase and microscopic analysis revealed that 22% of H2228 (V3) cells had lagging chromosomes compared with 17% of H3122 (V1) cells in anaphase (Fig. 2E and F). Lagging chromosomes are often not incorporated into the nucleus of a daughter cell and reside as micronuclei in interphase. Consistent with the higher proportion of lagging chromosomes, H2228 cells had significantly more micronuclei (20%) compared with H3122 cells (8%; Fig. 2G and H).

To confirm that this increased frequency of mitotic errors is not a result of background mutations in these NSCLC patient-derived cell lines, Beas-2B inducible cells expressing HA-EML4-ALK V1 and HA-EML4-ALK V3 were analyzed. Following 48 hours induction with doxycycline, it was found that the proportion of multipolar spindles and misaligned chromosomes in metaphase, lagging chromosomes in anaphase and micronuclei in interphase were significantly higher in cells expressing HA-EML4-ALK V3 compared with either cells expressing HA-EML4-ALK V1 or parental Beas-2B cells (Supplementary Figs. S2A–S2H). In addition, the proportion of interphase cells with amplified centrosomes suggestive of cell division defects was elevated for both H2228 and Beas-2B:HA-EML4-ALK V3 cells when compared with H3122 and Beas-2B:HA-EML4-ALK V1 (or parental Beas-2B) cells, respectively (Supplementary Figs. S2I–S2L). Taken together, these data indicate that expression of EML4-ALK V3 but not V1 significantly increases mitotic errors, which in turn may promote genome instability.

### EML4-ALK V3 localizes to microtubules of the mitotic spindle

**Figure 3.**
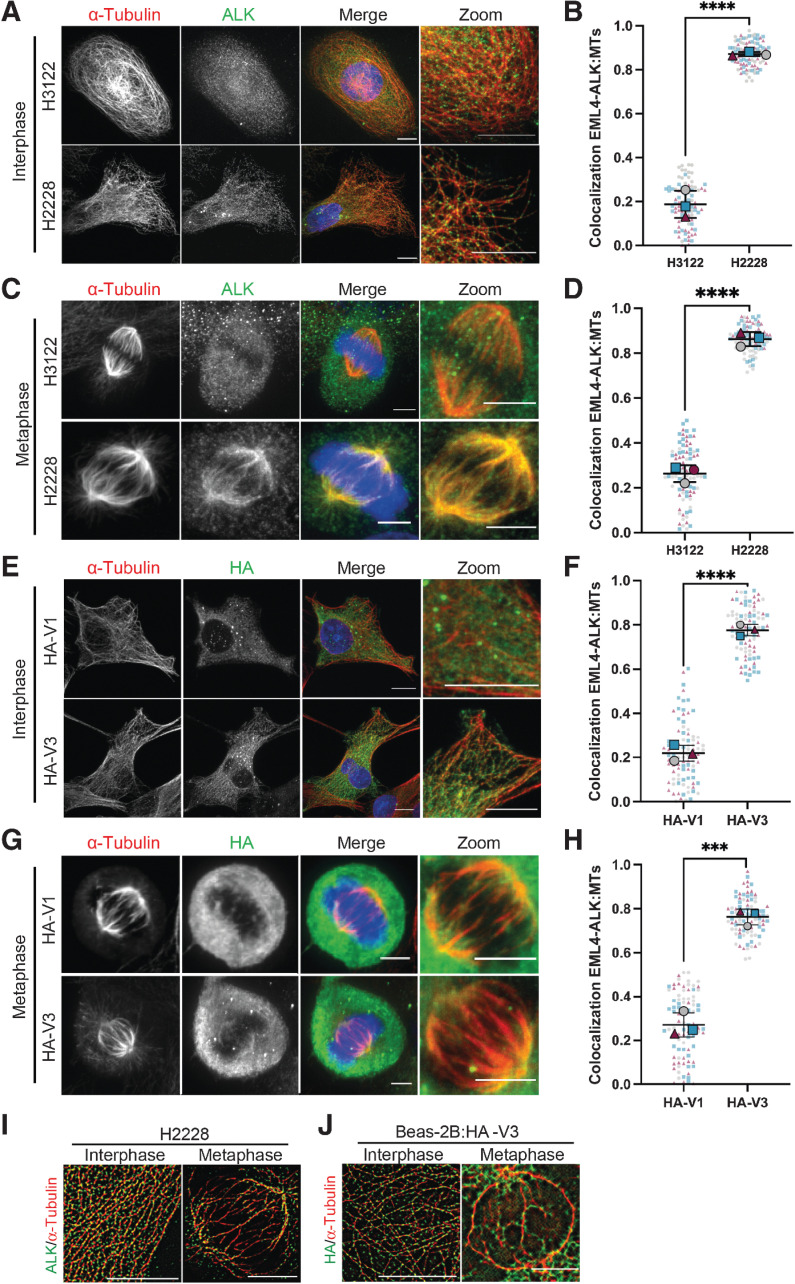
EML4-ALK V3 but not V1 localizes to microtubules during mitosis. **A–D,** H2228 and H3122 cells were grown on coverslips, stained with α-tubulin (red) and ALK (green) antibodies, and subjected to immunofluorescence microscopy analysis. DNA was stained with Hoechst 33258 (blue). Scale bars in metaphase, 5 μm, and in interphase, 10 μm. Colocalization of EML4-ALK with microtubules in interphase (**A** and **B**) and metaphase (**C** and **D**) was determined by measuring Pearson correlation coefficient along three 2 µm lines in 10 cells. ****, *P* < 0.0001 compared with H3122 cells by unpaired *T* test. **E–H,** Beas-2B cells expressing either HA-EML4-ALK V3 or V1 were induced for 48 hours and stained with HA (green) and α-tubulin (red) antibodies. DNA was stained with Hoechst 33258 (blue). Colocalization of EML4-ALK with microtubules in interphase (**E** and **F**) and metaphase (**G** and **H**) was determined by measuring Pearson correlation coefficient along three 2 µm lines in 10 cells. ****, *P* < 0.0001 and ***, *P* < 0.001 compared with HA-V1 cells by unpaired *T* test. Scale bars in metaphase, 5 μm, and in interphase, 10 μm. The three different shapes and colours in the dot plots in **B, D, F,** and **H** indicate data from three independent experiments, with large shapes referring to means and small shapes the individual data points. **I** and **J,** SRRF image reconstruction of EML4-ALK V3 (green) microtubules (red) in an interphase and metaphase H2228 (**I**) and Beas-2B:HA-EML4-ALK V3 (**J**) cell stained as above. Data are means +SD for three independent experiments.

Wild-type EML4 localizes as punctate foci along the outer surface of microtubules in interphase but is displaced from microtubules in mitosis as a result of phosphorylation; meanwhile EML4-ALK V3 but not V1 localizes to microtubules in interphase cells (8, 18, 20, 22). Here, we compared the interphase and mitotic localization of EML4-ALK V1 and V3 in NSCLC patient-derived cell lines as well as isogenic Beas-2B cells by confocal microscopy. Cells were stained with ALK and α-tubulin antibodies and the Pearson correlation coefficient (*R*) of colocalization between the EML4-ALK protein and microtubules determined on the basis of lines drawn on individual z-sections. These analyses revealed substantially stronger association of EML4-ALK V3 with interphase microtubules in H2228 cells (*R* = 0.87 ± 0.009) than H3122 cells (*R* = 0.22 ± 0.07), as described previously (Fig. 3A and B; ref. 20). Unexpectedly though, analyses of metaphase cells revealed that EML4-ALK V3 protein remained strongly associated with spindle microtubules in H2228 cells (*R* = 0.86 ± 0.03), whereas EML4-ALK V1 exhibited little association with microtubules in H3122 cells (*R* = 0.18 ± 0.06; Fig. 3C and D). Examination of the localization of HA-EML4-ALK V1 and V3 following doxycycline induction in Beas-2B cells confirmed that the V3 (*R* = 0.78 ± 0.02) but not V1 (*R* = 0.22 ± 0.03) protein associated with microtubules in interphase (Fig. 3E and F). Furthermore, as observed in the ALK-positive NSCLC-derived cells, the HA-tagged V1 protein exhibited disperse cytoplasmic localization in metaphase Beas-2B cells with little association to microtubules (*R* = 0.27 ± 0.05), whereas HA-V3 exhibited strong spindle microtubule localization (*R* = 0.76 ± 0.03; Fig. 3G and H).

To gain a higher resolution view of EML4-ALK V3 protein localization on microtubules, super-resolution radial fluctuation (SRRF) imaging was carried out on H2228 cells and Beas-2B cells expressing HA-EML4-ALK V3. This revealed that the EML4-ALK V3 protein formed multiple small discrete puncta that were evenly distributed along the lengths of interphase microtubules and spindle fibers without obvious concentration at microtubule ends (Fig. 3I and J). These were clearly distinct to a small number of larger cytoplasmic foci of EML4-ALK observed in both the NSCLC and recombinant Beas-2B V1 and V3 cell lines that were consistent with the previously documented phase-separated liquid droplets (21–23). Taken together, these data indicate that the EML4-ALK V3 but not V1 protein is strongly associated with microtubules throughout the cell cycle and is retained on spindle fibers in mitosis.

### Expression of EML4-ALK V3 leads to compromised SAC activity

**Figure 4.**
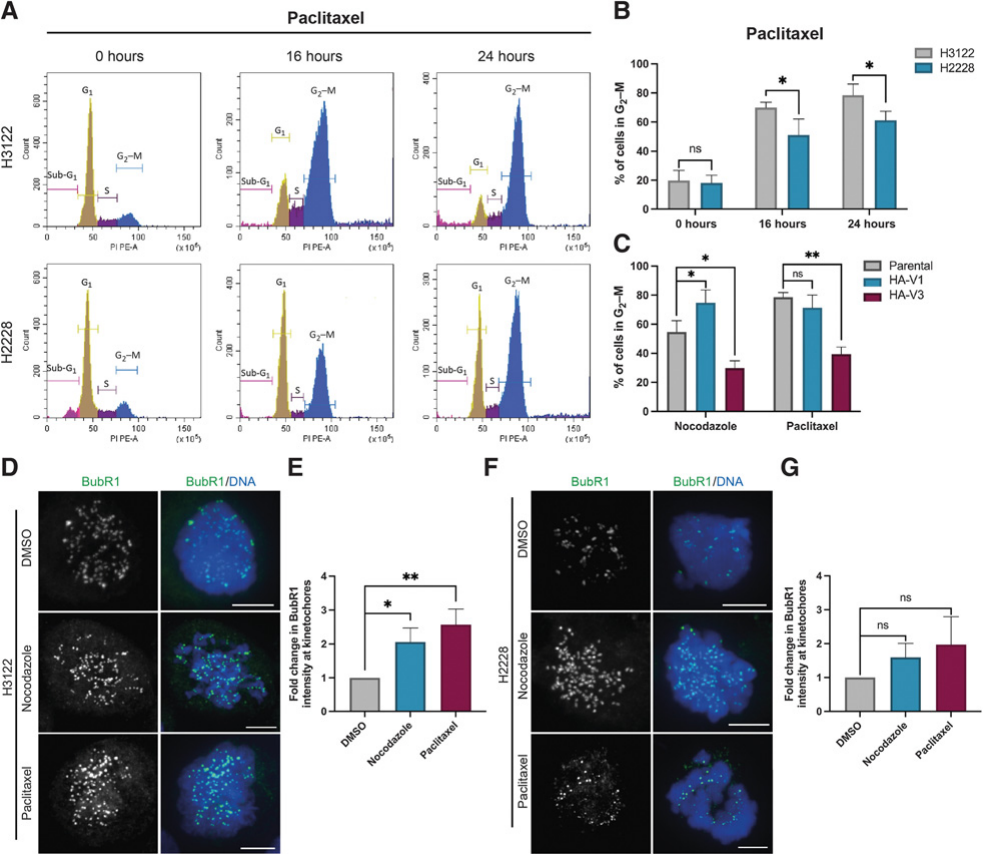
EML4-ALK V3 expressing cells have compromised SAC activity. **A,** H3122 and H2228 cells were treated with 5 μmol/L paclitaxel for 0, 16, or 24 hours and cell-cycle distribution quantified by staining with propidium iodide and cell-cycle distribution measured using flow cytometry. **B,** The percentage of H3122 (gray bars) and H2228 (blue bars) cells in G_2_–M based on analyses performed in **A** is shown. *, *P* < 0.05 compared with H3122 by unpaired *T* test. **C,** Beas-2B parental (gray) and HA-EML4-ALK V1 (blue) and HA-V3 (red) cells were induced with doxycycline for 48 hours and treated with 0.5 μmol/L nocodazole or 5 μmol/L paclitaxel for 24 hours and the percentage of cells in G_2_–M quantified using flow cytometry. *, *P* < 0.05, **P < 0.01 compared with parental by one-way ANOVA. **D,** H3122 cells were treated with DMSO alone, 0.5 μmol/L nocodazole or 5 μmol/L paclitaxel for 16 hours and stained with BubR1 (green) antibodies and Hoechst 33258 (blue). Scale bars, 5 μm. **E,** BubR1 intensity at kinetochores was quantified from the images shown in D using ImageJ and presented as fold-change compared with DMSO alone. *, *P* < 0.05 and **, *P* < 0.01 compared with DMSO by one-way ANOVA. **F** and **G,** Analyses were performed as in **D** and **E**, except using H2228 cells. Scale bars, 5 μm. Data are means +SD for three independent experiments.

Because of the significant increase in lagging chromosomes observed during anaphase in cells expressing EML4-ALK V3, the activity of the spindle assembly checkpoint (SAC) was analyzed. Using flow cytometry to investigate cell-cycle distribution, it was first noted that untreated H3122 and H2228 cells exhibit similar cell-cycle profiles with ∼18% to 20% cells in the G_2_–M phase of the cell cycle (Supplementary Figs. S3A and S3B). However, upon treatment with the microtubule poisons, paclitaxel (5 µmol/L) or nocodazole (0.5 µmol/L), there were significantly fewer H2228 (V3) cells arrested in G_2_–M than H3122 (V1) cells (Fig. 4A and B; Supplementary Figs. S3C and S3D). Similarly, Beas-2B cells expressing HA-EML4-ALK V3 cells exhibited a less robust G_2_–M arrest following nocodazole or paclitaxel treatment compared with parental Beas-2B cells or Beas-2B cells expressing HA-EML4-ALK V1 (Fig. 4C). Interestingly, comparison of the isogenic Beas-2B cells revealed that expression of V3 but not V1 reduced the proportion of cells in the G_2_–M phase even in the absence of drug treatment (Supplementary Figs. S3E and S3F).

To further examine SAC integrity, quantitative imaging was performed of the SAC component, BubR1, at the kinetochores of metaphase spindles. Strikingly, this revealed that although there was an expected recruitment of BubR1 to kinetochores in metaphase following nocodazole or paclitaxel treatment in H3122 cells (Fig. 4D and E), there was reduced and nonsignificant recruitment of BubR1 to kinetochores in H2228 cells (Fig. 4F and G). Meanwhile, in the absence of drug treatment, H2228 cells had approximately three-fold less BubR1 at kinetochores than H3122 cells in metaphase (Supplementary Figs. S4A and S4B). Furthermore, Beas-2B cells expressing HA-EML4-ALK V3 had reduced staining of BubR1 in metaphase and progressed more rapidly through mitosis based on time-lapse imaging compared with parental cells or cells expressing HA-EML4-ALK V1 (Supplementary Figs. S4C–S4F). Considering that SAC activity is important to control mitotic progression under both normal conditions and when microtubule dynamics are perturbed, these data indicate that the SAC is significantly weakened in cells expressing EML4-ALK V3.

### Mitotic defects in cells expressing EML4-ALK V3 are independent of ALK activity

To determine whether the mitotic defects observed in cells expressing EML4-ALK V3 are a result of inappropriate ALK signaling, H2228 (V3) and Beas-2B HA-EML4-ALK V3 cells were treated with the experimental ALK tyrosine kinase inhibitor, TAE-684 (28). For these experiments, the concentration of TAE-684 used was determined by performing a cell viability dose response assay that indicated an IC_50_ value of 0.149 μmol/L for H2228 cells and 0.305 μmol/L for Beas-2B:HA-EML4-ALK V3 cells (Supplementary Fig. S5A). Using this IC_50_ dose, inhibition of downstream ALK signaling was confirmed in these two cell lines by Western blotting with pERK antibodies (Supplementary Figs. S5B and S5C). Immunofluorescence microscopy of H2228 cells treated with 0.149 μmol/L TAE-684 revealed that the proportion of cells with multipolar spindles, misaligned chromosomes, lagging anaphase chromosomes and micronuclei was not significantly altered (Supplementary Figs. S5D–S5K). Similarly, treatment of Beas-2B:HA-EML4-ALK V3 cells with 0.305 μmol/L TAE-684 did not reduce the frequency of any of the measured mitotic defects other than multipolar spindles (Supplementary Figs. S5L–S5S). The reason for the reduced multipolar spindles is unclear although could reflect some off-target activity of TAE-684 in these cells (29). However, taken together, these data suggest that the mitotic defects observed in cells expressing EML4-ALK V3 are largely independent of ALK signaling.

### Loss of SAC function in EML4-ALK V3 cells is partly dependent on ALK activity

**Figure 5.**
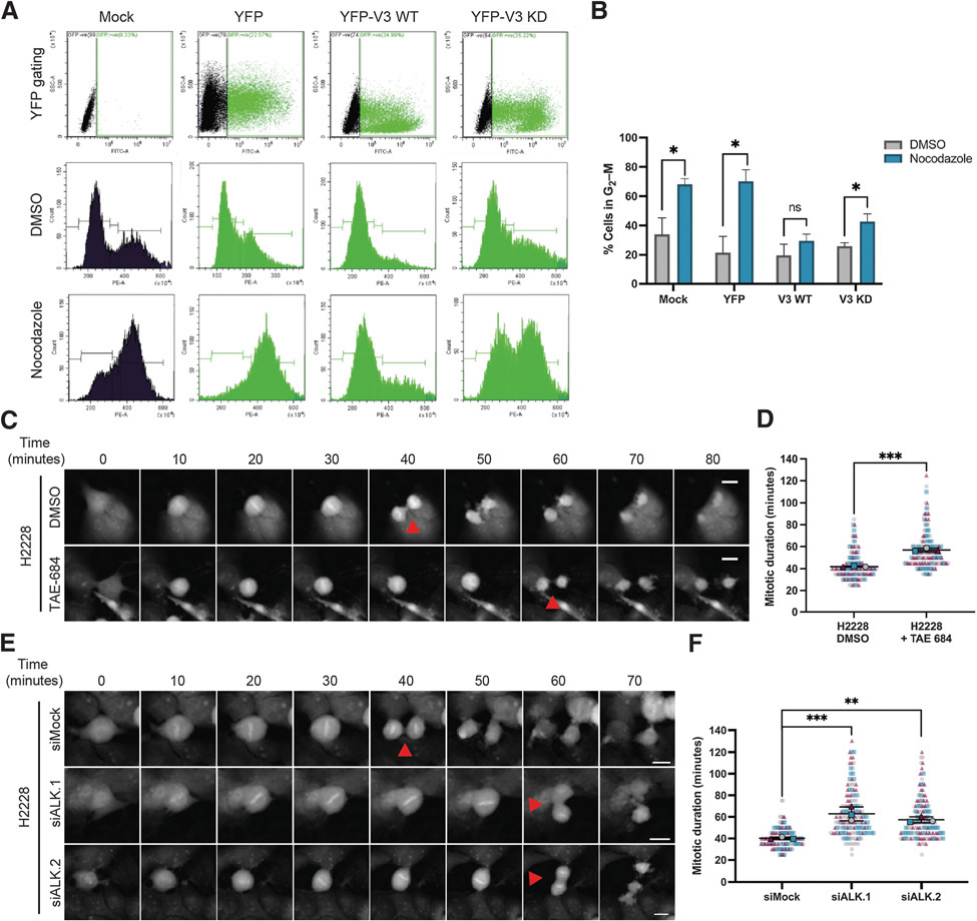
SAC deficiencies in cells expressing EML4-ALK V3 are dependent on ALK activity. **A,** Beas-2B parental cells were either mock transfected or transfected with plasmids encoding YFP alone, YFP-EML4-ALK V3 WT (wild-type) or YFP-EML4-ALK V3 KD (kinase-dead) for 24 hours. They were then treated with either DMSO alone or 0.5 µmol/L nocodazole for 16 hours before being analysed by flow cytometry. The top row indicates how cells were gated for expression of YFP using green channel forward scatter with the gate set above the upper threshold of fluorescence in the mock-transfected cells. The middle and bottom rows indicate DNA content following treatment with DMSO or nocodazole, respectively, with the profile shown for the ungated mock-transfected cells but the gated cells for those transfected with the different plasmids. **B,** The percentage of cells in G_2_–M were determined following treatment described in A. *, *P* < 0.05 compared with DMSO by unpaired T-test. **C,** H2228 cells were treated with DMSO alone or 0.149 µmol/L TAE-684 and analysed by time-lapse imaging; images captured at the times indicated (minutes) are shown. **D,** Quantification of mitotic duration for cells treated as in **C**. ***, *P* < 0.001 compared with DMSO by unpaired *T* test. **E,** H2228 cells were either mock-depleted or depleted of EML4-ALK using siALK.1 or siALK.2 and analysed as in **C**. **F,** Quantification of mitotic duration for cells treated as in **E**. **, *P* < 0.01 and ***, *P* < 0.001 compared with siMock by one-way ANOVA. Data are means +SD for three independent experiments (indicated in different colours in **D** and **F**). The three different shapes and colors in the dot plots in **D** and **F** indicate data from three independent experiments, with large shapes referring to means and small shapes the individual data points. Scale bars, 20 μm.

To investigate whether the loss of SAC activity in cells expressing EML4-ALK V3 is dependent on ALK activity, Beas-2B cells were first either mock transfected or transfected with plasmids expressing YFP alone, YFP-EML4-ALK V3 or a kinase-dead mutant (D1270N) of YFP-EML4-ALK V3 before incubation with either DMSO or nocodazole. As expected, mock transfected and YFP transfected Beas-2B cells showed significant G_2_–M arrest upon nocodazole treatment (Fig. 5A and B). In contrast, cells transfected with wild-type V3 displayed no significant increase in G_2_–M after nocodzole treatment consistent with V3 interfering with SAC activity. However, cells transfected with kinase-dead V3 displayed an intermediate level of G_2_–M arrest upon nocodazole treatment that was significantly increased compared with DMSO alone but not to the same extent as the mock or YFP-transfected cells. Next, either parental Beas-2B or Beas-2B:HA-EML4-ALK V3 cells were induced for 48 hours with doxycycline and then treated for 24 hours with either nocodazole, TAE-684, or a combination of both drugs and cell-cycle distribution analyzed by flow cytometry (Supplementary Fig. S6A). The Beas-2B parental cells exhibited a substantial G_2_–M arrest in response to treatment with nocodazole (or a combination of nocodazole and TAE-684) but not TAE-684 alone (Supplementary Fig. S6B). In contrast, Beas-2B cells expressing HA-EML4-ALK V3 exhibited substantially reduced G_2_–M arrest with nocodazole alone or in combination with TAE-684 consistent with loss of the SAC. Interestingly though, treatment of these cells with TAE-684 led to a small increase in the proportion of cells in G_2_–M compared with DMSO treatment, whereas the combination of TAE-684 and nocodazole also led to an increase in the G_2_–M population compared with nocodazole alone. These results together support the hypothesis that ALK catalytic activity does make some contribution to SAC function although other consequences of EML4-ALK V3 expression are also likely to be important.

To further investigate the impact of ALK inhibition on mitotic progression in EML4-ALK V3 cells, H2228 cells were treated with 0.149 μmol/L TAE-684 and analyzed by time-lapse imaging. Quantification confirmed previous data that inhibition of ALK catalytic activity significantly increased mitotic duration in H2228 cells (Fig. 5C and D; ref. 30). Finally, it was found that the mitotic duration of H2228 cells was significantly increased following depletion of EML4-ALK V3 compared with mock depletion (Fig. 5E and F). Taken together, these data indicate that expression of EML4-ALK V3 perturbs SAC integrity in a manner that is at least partly dependent on ALK activity.

### Synergistic response of H2228 cells expressing EML4-ALK V3 to treatment with paclitaxel and targeted ALK inhibitors

**Figure 6.**
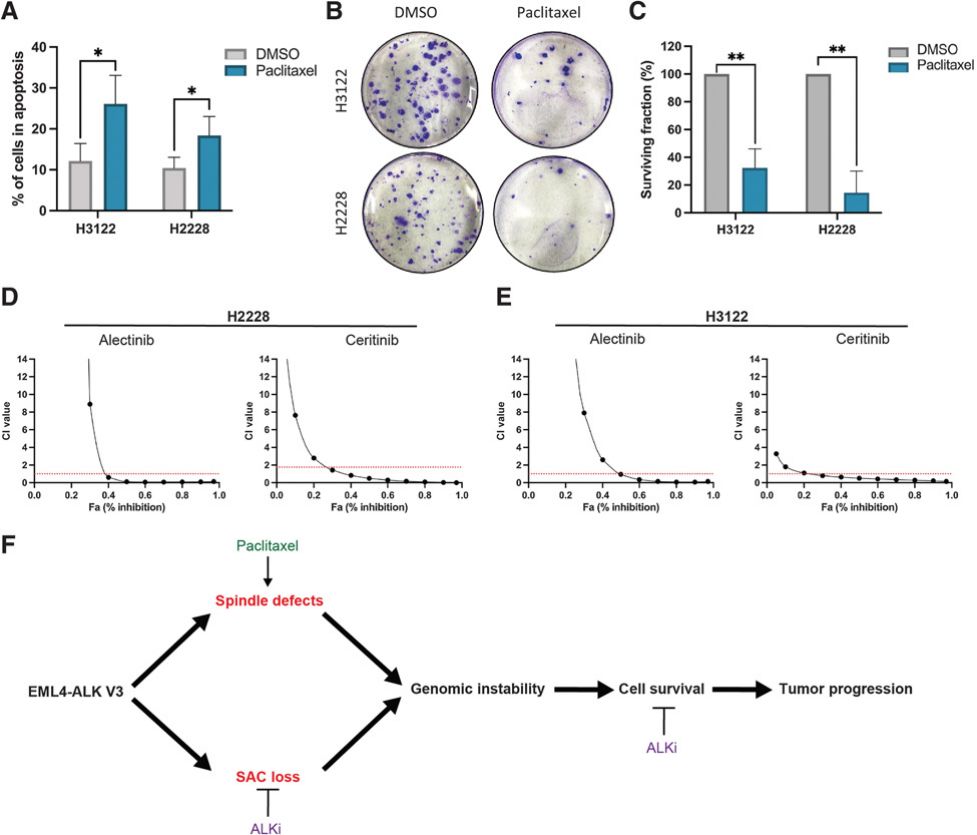
Synergistic response of H3122 and H2228 cells following combination treatment with paclitaxel and ALK inhibitors. **A,** H3122 and H2228 cells were treated with either DMSO or 4.27 µmol/L paclitaxel for 24 hours and the percentage of cells undergoing apoptosis determined by Annexin-V-PI staining and flow cytometry. *, *P* < 0.05 compared with DMSO by unpaired *T* test. **B,** H3122 and H2228 cells were treated with DMSO alone or 4.27 µmol/L paclitaxel for 24 hours before being replated at low density and cultured for 2 weeks to allow colonies to form. Colonies were detected by crystal violet staining. **C,** The surviving fraction of cells was calculated from the clonogenic assays described in **B**. **, *P* < 0.01 compared with DMSO by unpaired *T* test. **D** and **E,** Cell viability was quantified using Alamar blue following combination treatment of paclitaxel and alectinib or ceritinib at 1:1 ratios over a range of concentrations (1, 10, 100, 1,000, 10,000 nmol/L) using alamar blue and computed as the fraction effected (Fa, % inhibition of cell viability) compared with DMSO treated controls. Compusyn was used to generate Fa%: CI graphs for H2228 (**D**) and H3122 (**E**). **F,** This schematic model illustrates how expression of EML4-ALK V3, which induces both mitotic defects and loss of SAC activity, may promote genome instability, cell survival, and tumor progression. Combination treatment with ALK inhibitors, which support SAC activity, and paclitaxel, which also induces mitotic errors, may lead to improved outcomes for patients with ALK-positive NSCLC.

As cells expressing EML4-ALK V3 have reduced SAC activity, including in response to the microtubule stabilizing drug paclitaxel, the effect of combining clinically relevant targeted ALK inhibitors with paclitaxel on apoptosis and cell viability was examined in the patient-derived ALK-positive NSCLC cells. First, H3122 and H2228 cells were treated for 24 hours with 4.27 μmol/L paclitaxel, which is approximately equivalent to the *C*_max_ achievable in patients (31). Analysis by flow cytometry with Annexin-V staining revealed that the percentage of cells undergoing apoptosis increased from ∼10% with DMSO alone to 25% (for H3122) and 19% (for H2228) following treatment with paclitaxel (Fig. 6A). In addition, there was a substantial reduction in long-term viability of H3122 and H2228 cells following 24 hours of paclitaxel treatment as determined by clonogenic assays (Fig. 6B and C). In preparation for testing combination treatments with ALK inhibitors, we next determined the IC_50_ concentrations of alectinib, ceritinib, and crizotinib in H3122 and H2228 cells based on cell viability assays and confirmed that these doses led to loss of ALK-dependent signaling (Supplementary Figs. S7A–S7D). We then demonstrated that combination of these ALK inhibitors with paclitaxel significantly increased apoptosis (Supplementary Figs. S7E–S7J).

To investigate potential synergy between paclitaxel and targeted ALK inhibitors, the viability of H3122 and H2228 cells was analyzed in the presence of combinations of these drugs. The Chou–Talalay method was used to calculate the combination index (CI) of synergy in which the CI value is >1 when two drugs are antagonistic, 0.9 to 1 when they are additive, and <0.9 if they are synergistic (24, 25). First, cell viability data from H2228 and H3122 cells treated with 1:1 ratios of paclitaxel with alectinib and ceritinib (e.g., 1 nmol/L paclitaxel and 1 nmol/L ALK TKI) over a range of concentrations of drugs (1, 10, 100, 1,000, 10,000 nmol/L) were used to generate CI values according to the fraction affected (Fa) in terms of inhibition of cell viability (Fig. 6D and E). These data indicate that in H2228 cells, alectinib and ceritinib in combination with paclitaxel are highly synergistic with a CI value (at 0.5 Fa of the TKI) of 0.11 for alectinib and 0.5 for ceritinib. In H3122 cells alectinib and paclitaxel were additive as shown by a CI value of 0.95 whereas ceritinib and paclitaxel were synergistic (CI, 0.51). Further to this, alternative ratios of ALK TKI (alectinib, ceritinib, and crizotinib) with paclitaxel (1:10, 10:1, 100:1, 1:100) were investigated (Supplementary Fig. S8). These data indicated that at the majority of ratios, combination treatments were synergistic with CI values <0.9 at Fa 0.5 (Table 1). Interestingly, the number of synergistic combinations was higher in H2228 cells than H3122 cells. Taken together, these data demonstrate that H2228 cells that express EML4-ALK V3 are not only sensitive to paclitaxel alone but respond in a synergistic manner when paclitaxel is combined with clinically relevant ALK inhibitors.

**Table 1.**
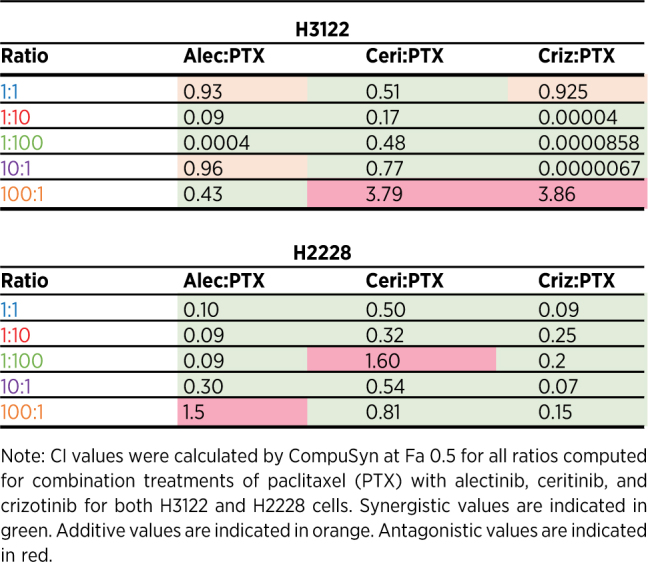
Summary of CI values at Fa 0.5 for H3122 and H2228 combination treatments.

## Discussion

The EML4-ALK fusion protein acts as an oncogenic driver in around 5% of NSCLC cases. Of these, patients with V3 display an elevated rate of metastasis, increased resistance to ALK inhibitors, and reduced overall survival compared with those with other variants (32, 33). Hence, a better understanding of the underlying biology of these tumors is urgently required so that alternative treatment strategies can be devised.

Here we demonstrate using patients with ALK-positive NSCLC cells and isogenic recombinant Beas-2B cells that expression of EML4-ALK V3 but not V1 leads to a range of mitotic errors, including formation of multipolar spindles, misaligned chromosomes, and lagging chromosomes. A higher frequency of micronuclei and amplified centrosomes were also observed in interphase cells expressing V3 that could reflect unequal segregation of chromosomes and centrosomes, as well as complete cell division failure. So what are the mechanisms that underlie these errors? We observed hyperstabilization of K-fibers in V3 expressing cells whereas depletion of V3 led to reduced intensity of both spindle and astral microtubules. Hence, these errors may well relate to the activity of the EML4 portion of the fusion protein, as EML4 is recognized as a microtubule stabilizing protein. Indeed, using a specific chemical inhibitor of ALK, TAE-684, we observed that the majority of these mitotic errors are not dependent on the catalytic activity of ALK. The fact that mitotic errors are not observed in cells expressing V1 also suggest that they do not arise as a consequence of downstream ALK signaling. Previous results indicated that a phospho-null mutant of full-length EML4 is not only retained on the spindle in mitosis but also promotes hyperstabilization of K-fibers and defects in chromosome congression (8). Therefore, these results are consistent with the inappropriate retention of the EML4-ALK V3 protein, via its N-terminal fragment, on microtubules, rather than ALK signaling, being a major cause of the mitotic errors observed. Furthermore, as EML4-ALK V3 can recruit the mitotic kinases NEK7 and NEK9 to microtubules, it is plausible that in addition to the increased stabilization of microtubules, aberrant localization of these kinases might contribute to the mitotic errors observed in V3 expressing cells (20).

The presence of mitotic errors in cells expressing V3 but not V1 correlates with localization of the V3 but not V1 protein to spindle microtubules. Indeed, the extent of microtubule association of V3 was similar between interphase and mitosis. This is in contrast to the full-length EML4 protein, which is displaced from microtubules upon mitotic entry (8). The cell-cycle-dependent regulation of wild-type EML4 is at least in part the result of phosphorylation in mitosis of residues within the basic linker by the NEK6 and NEK7 kinases. The fact that EML4-ALK V3 is capable of binding NEK7 and its upstream regulator NEK9 suggests that the retention of V3 on spindle microtubules is not the result of an inability to interact with these regulatory kinases (20) and further studies will be required to understand why EML4-ALK V3 is retained on microtubules in mitosis. Interestingly, recent studies into EML4-ALK localization have indicated that active ALK signaling occurs in phase-separated liquid droplets (21–23). Treatment of these cells with ALK inhibitors promoted disruption of these liquid droplets with EML4-ALK V3 localization becoming more strongly associated with microtubules under these conditions (22). The fact that these droplets also contained downstream signaling components led to the hypothesis that active ALK signaling primarily occur in liquid droplets within the cytoplasm with the EML4-ALK V3 protein residing on microtubules being less active (22). This adds further weight to the notion that the mitotic errors induced by the V3 protein are not dependent on ALK activity.

In addition to errors in spindle organization and chromosome congression and segregation, we found that cells expressing EML4-ALK V3 failed to arrest in mitosis and recruited less BubR1 to kinetochores when treated with the microtubule poisons, nocodazole and paclitaxel. These results indicate that cells with EML4-ALK V3 have weakened SAC activity. Indeed, the hyperstabilization of microtubules would be expected to delay normal mitotic progression and yet live cell imaging indicated that Beas-2B cells expressing EML4-ALK V3 progressed through mitosis at a faster rate than cells not expressing this variant. The reasons underlying defective SAC activity are unclear, although it is worth noting that EML4 has previously been reported to promote recruitment of NUDC to the spindle and that NUDC in turn promotes PLK1 localization to the kinetochore (9, 34). Hence, it is possible that kinetochore assembly is disrupted in cells expressing EML4-ALK V3 as a result of altered microtubule dynamics and trafficking. However, in this case we found that ALK activity may be partly responsible for perturbation of the SAC as cells expressing a kinase-inactive form of EML4-ALK V3 had a more robust SAC response. Interestingly, previous work had determined that the catalytic activity of ALK is important for mitotic progression of neuronal SH-SY5Y cells, which express high levels of ALK, with either ALK inhibitor treatment or ALK depletion slowing down progression through mitosis (30). Moreover, it was shown that ALK inhibitors, such as ceritinib and crizotinib, induce an M-phase delay as a result of SAC activation not only in neuronal SH-SY5Y cells but also in H2228 cells (30). We also confirmed in H2228 cells that treatment with TAE-684 or depletion of EML4-ALK V3 prolonged mitosis compared with untreated cells supporting the notion that ALK activity suppresses SAC function.

The increased frequency of mitotic errors together with loss of SAC integrity in cells expressing EML4-ALK V3 enhances the potential for genome instability. Indeed, analyses of circulating tumor cells revealed high levels of chromosomal instability in ALK-positive NSCLC patients that were resistant to ALK inhibitor treatment, and it will be interesting to determine whether this correlates with the EML4-ALK variant expressed (35). Interestingly, H2228 cells have previously been identified as having a highly aneuploid nature (36). Moreover, the finding that cells expressing EML4-ALK V3 have increased propensity for genome instability is important from a treatment point of view. Strikingly, we found that treatment of EML4-ALK V3 expressing H2228 cells with combinations of paclitaxel, which also stabilizes microtubules, with clinically approved ALK inhibitors produced a synergistic response in terms of loss of cell viability. Combination treatments were shown to increase apoptotic cell death of both H3122 and H2228 cells, with H2228 cells responding synergistically with paclitaxel across a range of concentrations of alectinib, ceretinib and crizotinib. The mechanism for this synergy remains to be determined but may well be complex. As paclitaxel stabilizes spindle microtubules whereas ALK inhibitors delay mitotic progression, then one hypothesis is that combination treatment could increase spindle defects thereby promoting genomic instability above a critical threshold while also blocking ALK-dependent survival pathways (Fig. 6F).

Previous investigations have revealed synergy of ALK inhibitors with other classes of drug in cells expressing EML4-ALK fusions. For example, dasatinib (an ABL and SRC kinase inhibitor) produced a synergistic response with crizotinib in both H3122 and H2228 cells (37), whereas a STAT3 inhibitor, YHO-1701, exhibited synergy with alectinib, crizotinib, and ceritinib in H2228 cells (38). Other studies have found ceritinib and paclitaxel to be highly synergistic in the treatment of lung cancer cells with activation of the FAK even if they do not express EML4-ALK fusions (39). In conclusion, patients with EML4-ALK V3 driven cancers have an unmet clinical need and the work presented here provides additional evidence that a polytherapy approach combining ALK inhibitors with, in this case, paclitaxel could be effective and reduce the potential for acquired resistance.

## Supplementary Material

Supplementary Figure
