# Supplementary material for: EML4-ALK Variant 3 Promotes Mitotic Errors and Spindle Assembly Checkpoint Deficiency Leading to Increased Microtubule Poison Sensitivity
Source: Mol Cancer Res. 2022 Feb 25;20(6):854–66. doi: 10.1158/1541-7786.MCR-21-1010 (PMC9381094; doi:10.1158/1541-7786.MCR-21-1010)
Supplement: Supplementary Figure [file mcr-21-1010_supplementary_figures_1-8_supp1-8.pdf]

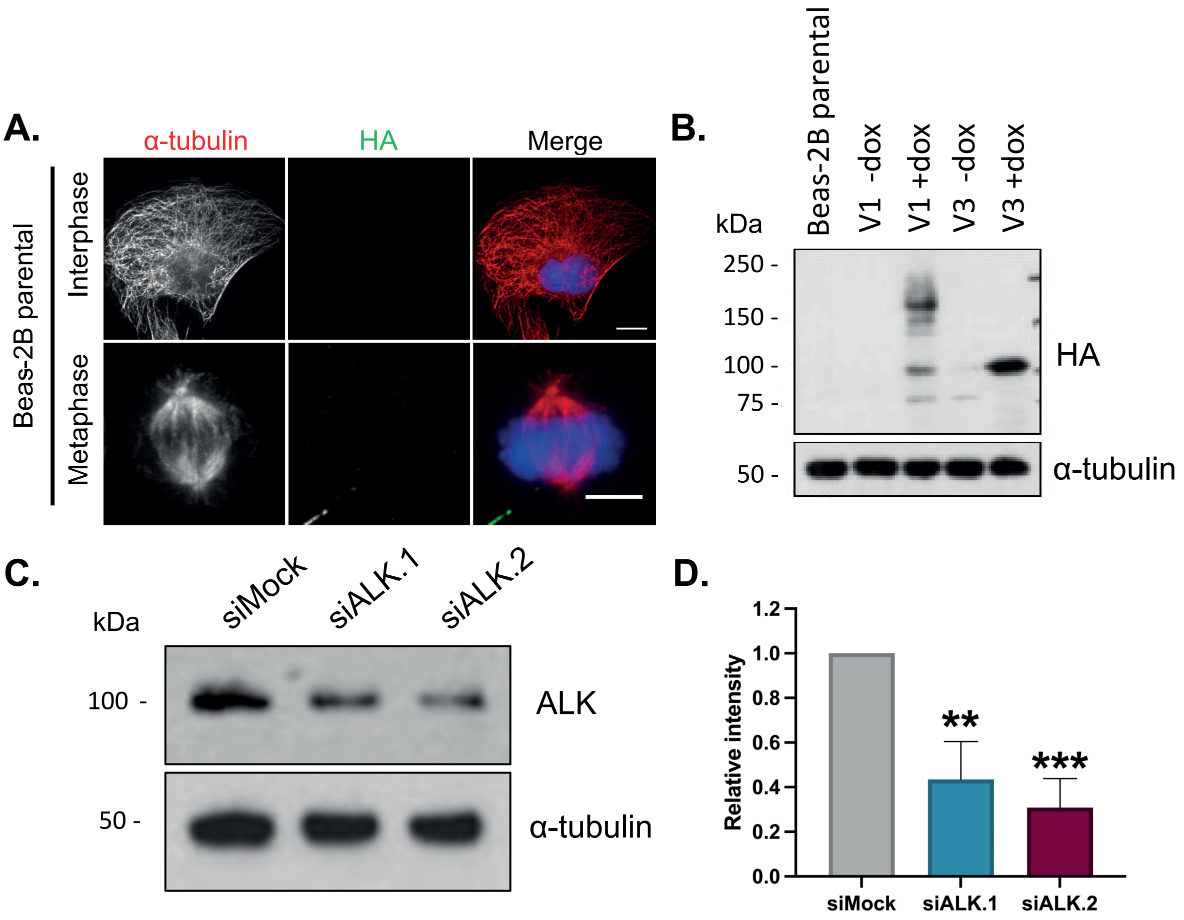

Supplementary Figure S1. HA-EML4-ALK protein expression in Beas-2B cells and depletion in H2228 cells.

A. Beas-2B parental cells were induced with doxycycline for 48 hours and stained with α-tubulin and HA antibodies. Scale bar interphase: 10 μm, metaphase: 5 μm. **B**. Western blot analyses were performed with HA and α-tubulin antibodies of lysates prepared from parental, HA-V1 and HA-V3 cells with (+dox) and without (-dox) 48 hours doxycycline induction. **C.** EML4-ALK V3 protein was depleted in H2228 cells using siMock or siALK oligonucleotides as indicated and Western blotted with ALK and α-tubulin antibodies. M. wt markers (kDa) are indicated on the left in B and C.
D. Densitometry quantification of Western blots shown in C. One-way ANOVA in comparison to siMock: **p<0.01, ***p<0.001. Data are means +SD for three independent experiments.

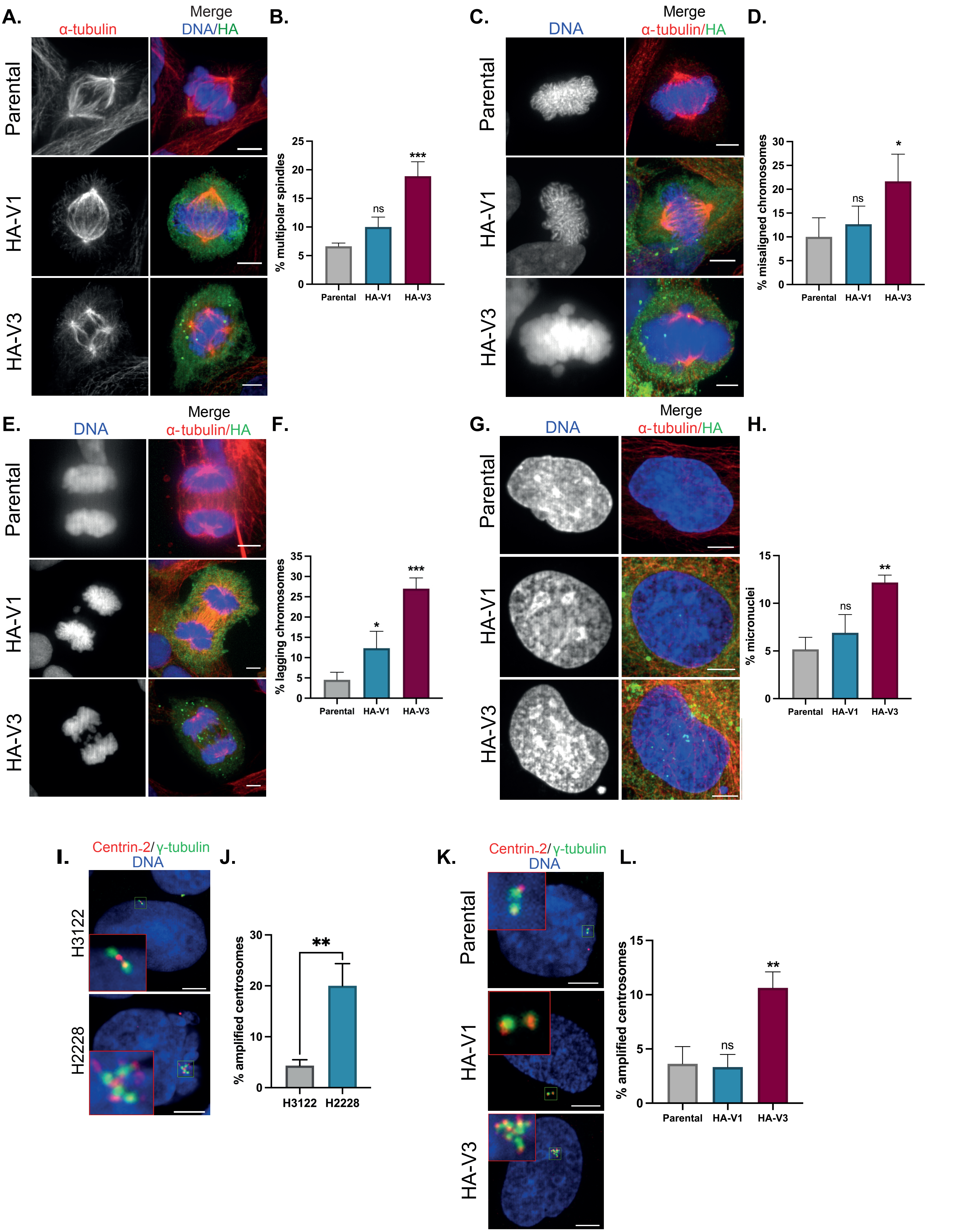
A.
α-tubulin
Merge
DNA/HA
Parental
HA-V1
HA-V3
B.
% multipolar spindles
0
5
10
15
20
25
ns
***
Parental
HA-V1
HA-V3
C.
DNA
Merge
α-tubulin/HA
Parental
HA-V1
HA-V3
D.
% misaligned chromosomes
0
5
10
15
20
25
30
ns
*
Parental
HA-V1
HA-V3
E.
DNA
Merge
α-tubulin/HA
Parental
HA-V1
HA-V3
F.
% lagging chromosomes
0
5
10
15
20
25
30
35
*
***
Parental
HA-V1
HA-V3
G.
DNA
Merge
α-tubulin/HA
Parental
HA-V1
HA-V3
H.
% micronuclei
0
5
10
15
ns
**
Parental
HA-V1
HA-V3
I.
Centrin-2/γ-tubulin
DNA
H3122
H2228
J.
% amplified centrosomes
0
10
20
30
**
H3122
H2228
K.
Centrin-2/γ-tubulin
DNA
Parental
HA-V1
HA-V3
L.
% amplified centrosomes
0
5
10
15
ns
**
Parental
HA-V1
HA-V3

Supplementary Figure S2. Cells expressing HA-EML4-ALK V3 exhibit multiple mitotic errors

A-H. Beas-2B parental, HA-EML4-ALK V1 and HA-EML4-ALK V3 cells were induced with doxycycline for 48 hours and processed for immunofluorescence microscopy with antibodies against HA (green) and α-tubulin (red); DNA was stained with Hoechst 33258 (blue). 100 cells were analysed and the percentage with multipolar spindles in metaphase (A, B), misaligned chromosomes in metaphase (C, D), lagging chromosomes in anaphase (E, F), and micronuclei in interphase (G, H) was quantified. *$p<0.05$, **$p<0.01$, ***$p<0.001$ in comparison with parental by one-way ANOVA (n.s., non-significant). **I**. H3122 and H2228 cells processed for immunofluorescence microscopy with antibodies against centrin-2 (red) and -tubulin (green); DNA was stained with Hoechst 33258 (blue). **J**. 100 interphase cells were analysed from cells stained as described in **I** and the percentage with amplified centrosomes quantified. **$p<0.01$ in comparison with parental by unpaired T-test. **K, L**. Beas-2B parental, HA-EML4-ALK V1 and HA-EML4-ALK V3 cells were induced with doxycycline for 48 hours and processed and analysed as in I and J **$p<0.01$ in comparison with parental by one-way ANOVA. Data are means +SD for three independent experiments. Scale bars, 5 μm.

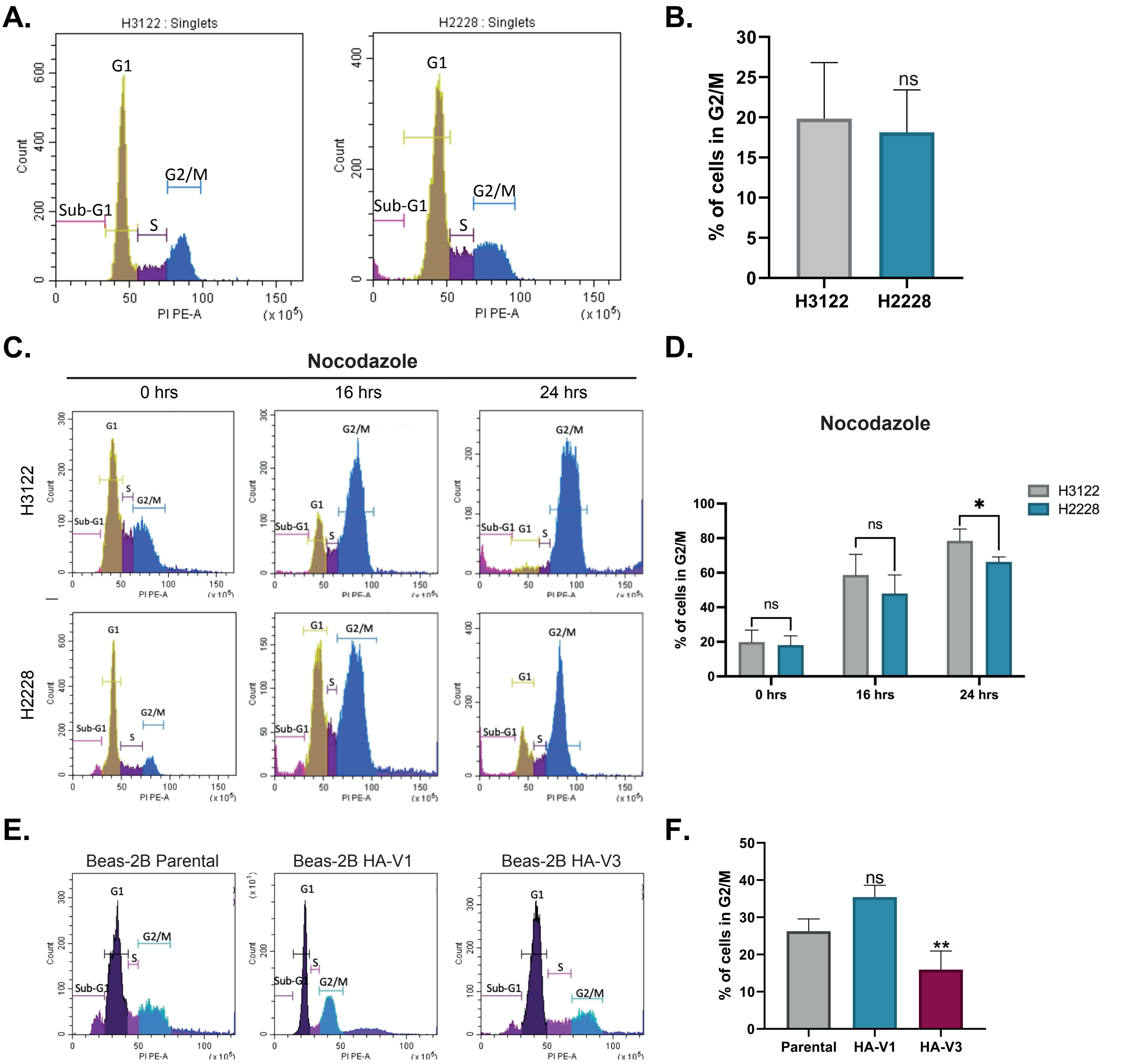

Supplementary Figure S3. H2228 cells expressing EML4-ALK V3 have reduced SAC activity compared to H3122
A, B. H3122 and H2228 cells were stained with propidium iodide to determine the percentage of cells in each phase of the cell cycle and analysed by flow cytometry. Cell cycle distribution (A) and percentage of cells in G2/M (B) are shown. **C, D**. H3122 and H2228 cells were treated with 0.5 μM nocodazole for 0, 16, or 24 hrs and analysed by flow cytometry. Cell cycle distribution (C) and percentage of cells in G2/M (D) are shown. *p<0.05 in comparison with H3122 by unpaired T-test. E, F. Beas-2B parental, HA-EML4-ALK V1 and V3 cells were induced with doxycycline for 48 hours before analysis by flow cytometry. Cell cycle distribution (**E**) and percentage of cells in G2/M (**F**) are shown. **p<0.01 in comparison with parental by one-way ANOVA. Data are means +SD for three independent experiments.

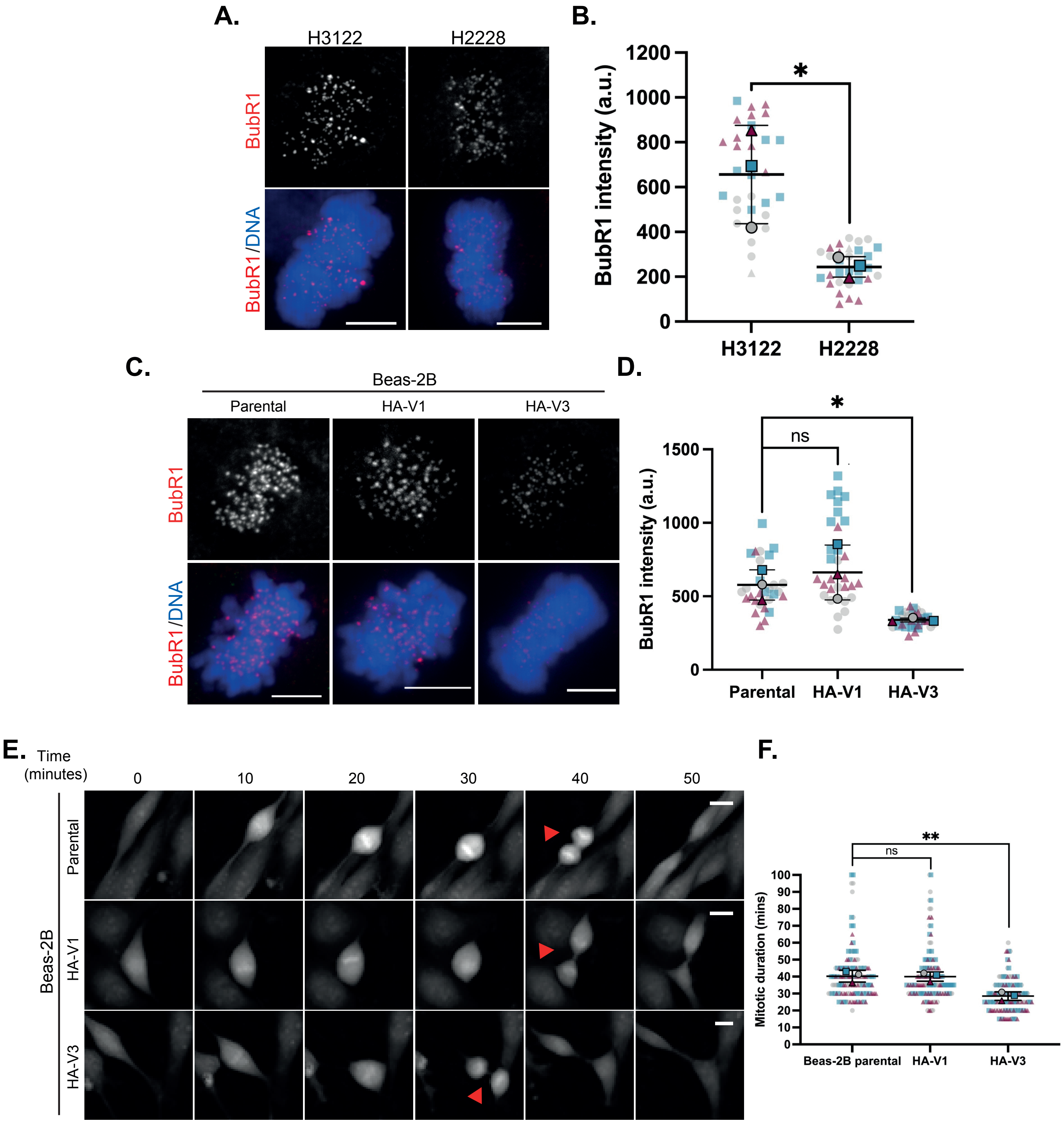
A.
H3122
H2228
BubR1
BubR1/DNA
B.
BubR1 intensity (a.u.)
*
H3122
H2228
C.
Beas-2B
Parental
HA-V1
HA-V3
BubR1
BubR1/DNA
D.
*
ns
BubR1 intensity (a.u.)
Parental
HA-V1
HA-V3
E.
Time (minutes)
0
10
20
30
40
50
Beas-2B
Parental
HA-V1
HA-V3
F.
**
ns
Mitotic duration (mins)
Beas-2B parental
HA-V1
HA-V3

Supplementary Figure S4. EML4-ALK V3 expressing cells have compromised SAC activity

A. H3122 and H2228 cells were processed for immunofluorescence microscopy with antibodies against BubR1 (red) and DNA stained with Hoechst 33258 (blue). **B.** The mean intensity of BubR1 staining from A was quantified by ImageJ analyses. *p<0.05 in comparison with H3122 by unpaired T-test. **C.** Beas-2B parental, HA-EML4-ALK V1 and V3 cells were induced with doxycycline for 48 hours and processed for immunofluorescence microscopy as in A. **D**. The mean intensity of BubR1 staining from C was quantified by ImageJ analyses. *p<0.05 in comparison with parental by unpaired one-way ANOVA. **E.** Beas-2B parental, HA-EML4-ALK V1 and V3 cells were induced with doxycycline for 48 hours and imaged by time-lapse microscopy every 5 mins for 24 hours. Examples of cells progressing through mitosis are indicated. Mitotic duration was calculated from the time cells rounded up to the point of cytokinesis (red arrows). **F**. Quantification of the duration of mitosis from cells in E. **p<0.01 in comparison with parental by unpaired one-way ANOVA. Data are means +SD for three independent experiments. The three different shapes and colours in the dot plots in B, D and F indicate data from three independent experiments, with large shapes referring to means and small shapes the individual data points. Scale bars in A and C, 5 µm, and 20 µm in E.

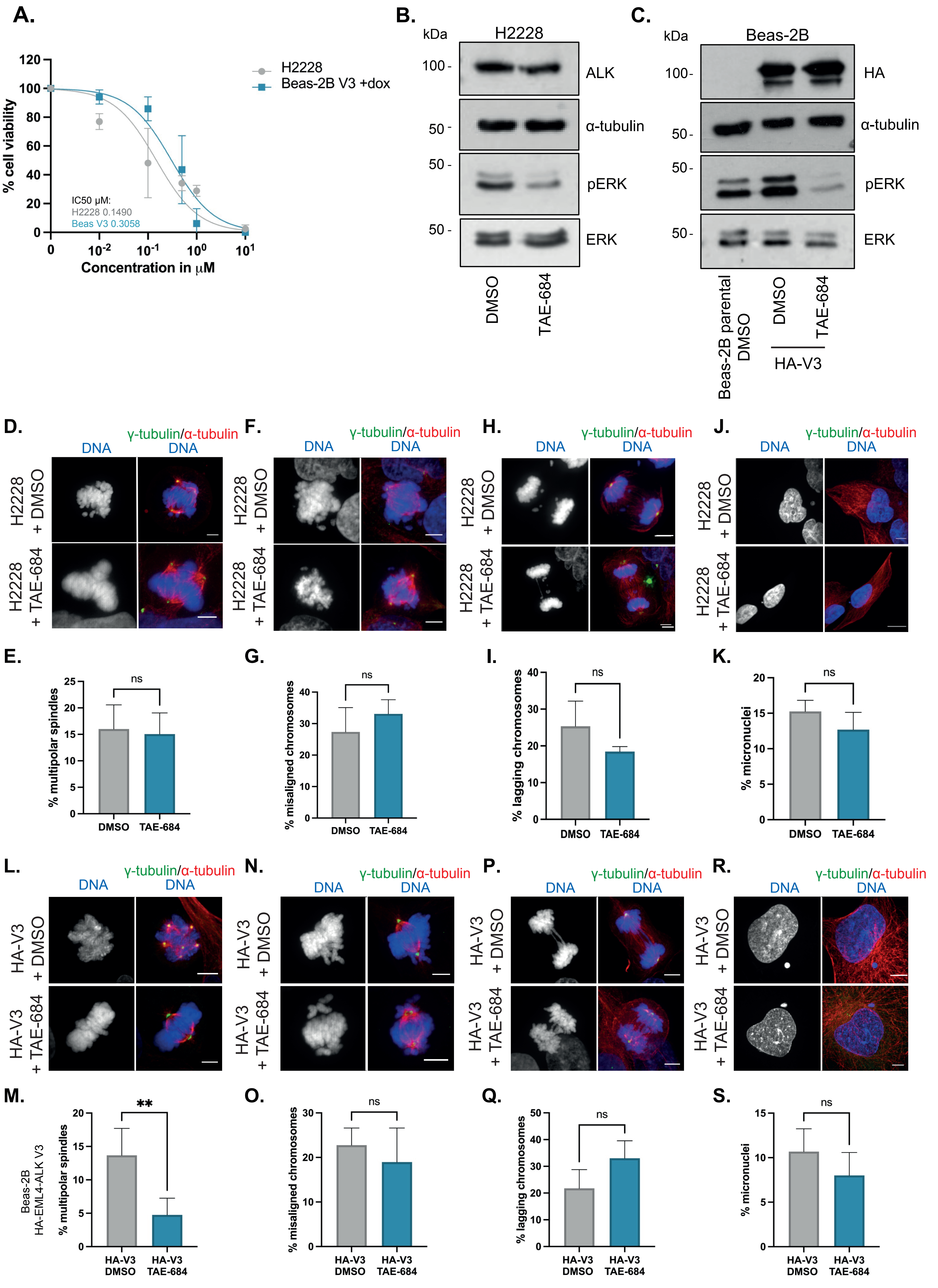
A.
% cell viability
Concentration in μM
H2228
Beas-2B V3 +dox
IC50 μM:
H2228 0.1490
Beas V3 0.3058
B.
kDa
H2228
ALK
α-tubulin
pERK
ERK
DMSO
TAE-684
C.
Beas-2B
HA
α-tubulin
pERK
ERK
Beas-2B parental DMSO
DMSO
TAE-684
HA-V3
D.
F.
H.
J.
DNA
γ-tubulin/α-tubulin DNA
H2228 + DMSO
H2228 + TAE-684
E.
% multipolar spindles
G.
% misaligned chromosomes
I.
% lagging chromosomes
K.
% micronuclei
ns
DMSO
TAE-684
L.
N.
P.
R.
HA-V3 + DMSO
HA-V3 + TAE-684
M.
Beas-2B HA-EML4-ALK V3
**
O.
Q.
S.
HA-V3 DMSO
HA-V3 TAE-684

Supplementary Figure S5. Mitotic defects in EML4-ALK V3 cells are independent of ALK activity

A. H2228 and Beas-2B HA-EML4-ALK V3 cells induced with doxycycline for 48 hours were treated with the indicated concentrations of TAE-684 for 48 hours and cell viability analysed by Alamar Blue assay. Calculated IC50 values are indicated. **B**. H2228 cells were treated with 0.149 μM TAE-684 for 6 hours and analysed by Western blotting with antibodies indicated. **C.** Parental Beas-2B and HA-EML4-ALK V3 cells were induced with doxycycline for 48 hours, treated with 0.3058 μM TAE-684 for 6 hours and cell lysates analysed by Western blotting with antibodies indicated. M. wts (kDa) are indicated on the left in B and C. **D-S**. H2228 cells treated with 0.1490 μM TAE-684 for 6 hours (**D-K**) or Beas-2B HA-EML4-ALK V3 cells induced with doxycycline for 48 hours and either untreated or treated with 0.3058 μM TAE-684 for 6 hours (**L-S**) were then stained for immunofluorescence microscopy with α-tubulin (red) and γ-tubulin (green) antibodies, and DNA stained with Hoechst 33258 (blue). 100 mitotic cells were analysed and the percentage of metaphase cells with multipolar spindles quantified (**D, E, L, M**). 100 metaphase cells were analysed and the percentage of cells with misaligned chromosomes quantified (**F, G, N, O**). 100 anaphase cells were analysed and the percentage of cells with lagging chromosomes quantified (**H, I, P, Q**). 100 interphase cells were analysed and the percentage of cells with micronuclei quantified (**J, K, R, S**). Data are means +SD for three independent experiments. Scale bars in mitosis, 5 μm, and interphase, 10 μm.

.

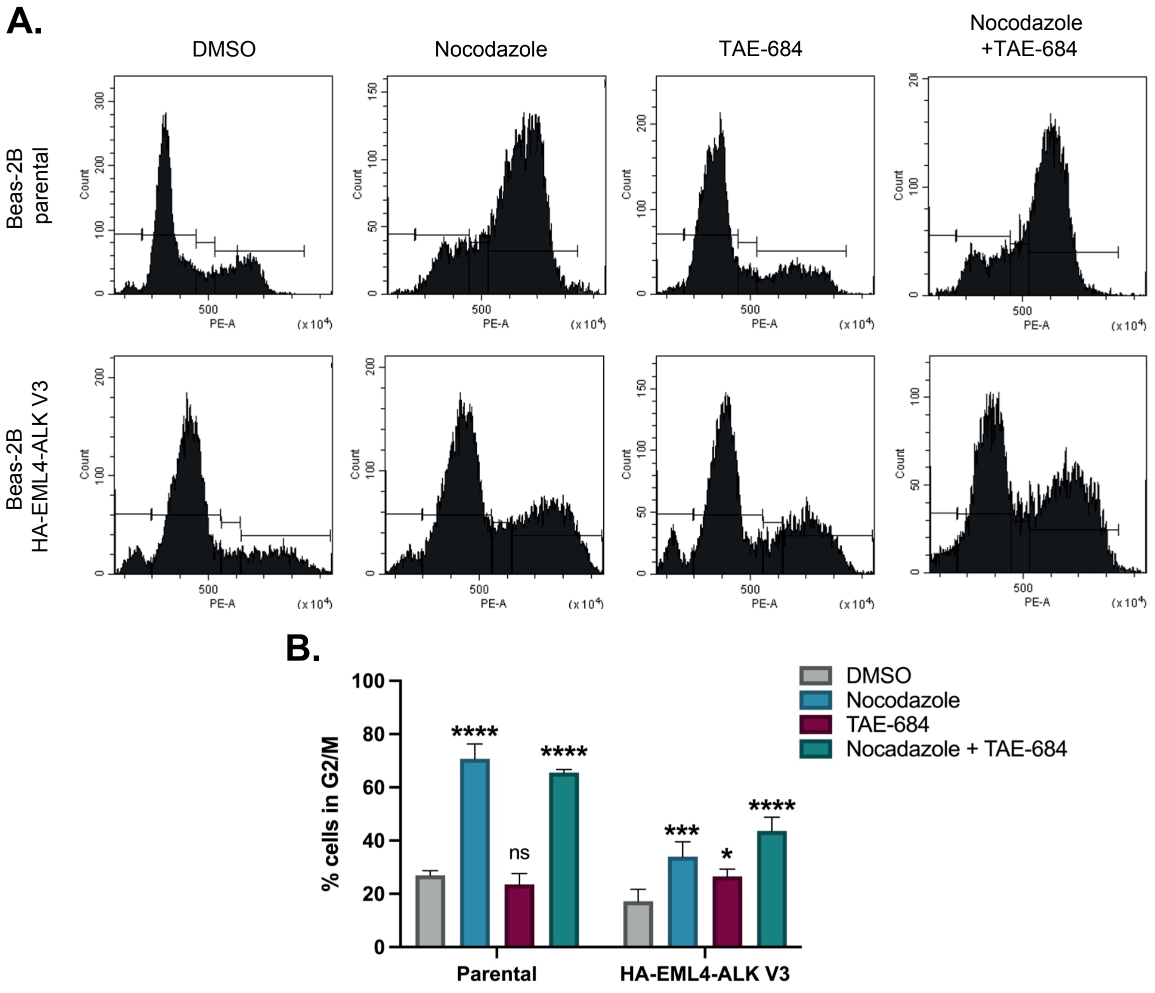

Supplementary Figure S6. HA-EML4-ALK V3 induced SAC deficiencies are partly dependent on ALK signalling activity

A. Beas-2B parental and HA-EML4-ALK V3 cells were induced with doxycycline for 48 hours and then treated with either DMSO alone, or 0.5 μM nocodazole or 0.3058 μM TAE-684 alone or in combination for 24 hours. Cells were analysed by flow cytometry and cell cycle profiles determined. B. The proportion of cells in G2/M from data in A was quantified. *p<0.05, ***p<0.001, ****p<0.0001 in comparison with DMSO by two-way ANOVA. Data are means +SD for three independent experiments.

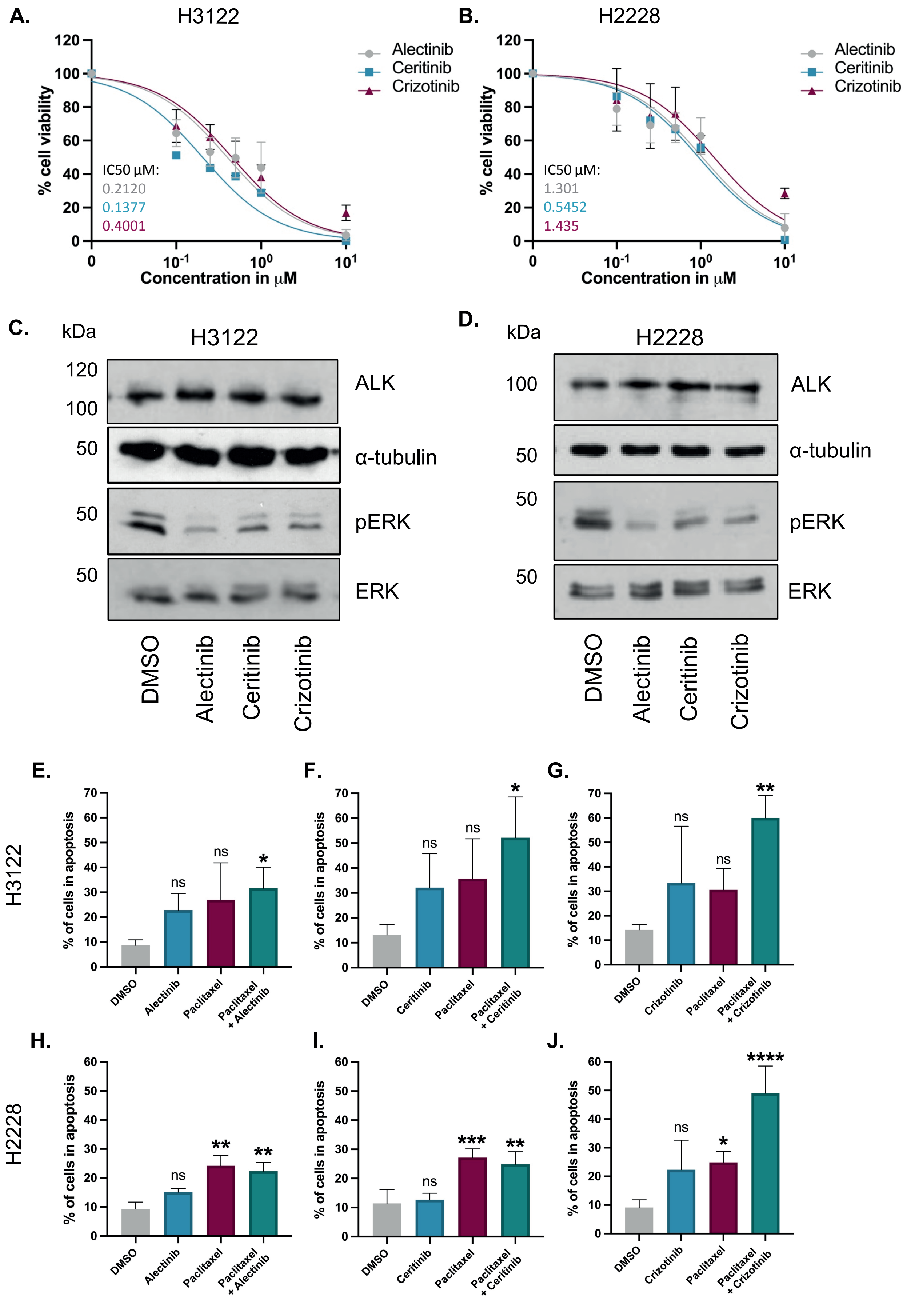

A.
H3122
% cell viability
Concentration in µM
IC50 µM:
0.2120
0.1377
0.4001
Alectinib
Ceritinib
Crizotinib
B.
H2228
% cell viability
Concentration in µM
IC50 µM:
1.301
0.5452
1.435
Alectinib
Ceritinib
Crizotinib
C.
kDa
H3122
ALK
α-tubulin
pERK
ERK
DMSO
Alectinib
Ceritinib
Crizotinib
D.
kDa
H2228
ALK
α-tubulin
pERK
ERK
DMSO
Alectinib
Ceritinib
Crizotinib
E.
F.
G.
H3122
% of cells in apoptosis
DMSO
Alectinib
Ceritinib
Crizotinib
Paclitaxel
Paclitaxel + Alectinib
Paclitaxel + Ceritinib
Paclitaxel + Crizotinib
H.
I.
J.
H2228
% of cells in apoptosis

Supplementary Figure S7. Combination treatment of H3122 and H2228 cells with paclitaxel and ALK inhibitors
A, B. H3122 (A) and H2228 (B) cells were treated with the indicated concentrations of alectinib, ceritinib and crizotinib for 48 hours and cell viability analysed by Alamar Blue assay. Calculated IC50 values are indicated. C, D. H3122 (C) and H2228 (D) cells were treated with IC50 concentrations of alectinib, ceritinib or crizotinib for 6 hours and cell lysates analysed by Western blotting with the antibodies indicated. M. wts (kDa) are indicated on the left. E-J. The percentage of H3122 or H2228 cells undergoing apoptosis following treatment for 48 hours with the ALK TKI indicated or paclitaxel alone or in combination was quantified by Annexin-V-FITC PI staining and flow cytometry. *p<0.05, **p<0.01, ***p<0.001, ****p<0.0001 in comparison with DMSO by one-way ANOVA. Data are means +SD for three independent experiments.

A.

H2228

Paclitaxel (nM) \ Alectinib (nM)	0	1	10	100	1000	10000
10000	0.79	0.84	0.88	0.87	0.92	0.97
1000	0.80	0.81	0.87	0.88	0.91	0.97
100	0.83	0.77	0.83	0.87	0.90	0.95
10	0.77	0.77	0.76	0.85	0.87	0.96
1	0.40	0.52	0.54	0.72	0.76	0.95
0	0.00	0.24	0.67	0.66	0.71	0.93

C.

H2228

Paclitaxel (nM) \ Ceritinib (nM)	0	1	10	100	1000	10000
10000	0.83	0.83	0.88	0.91	0.95	0.99
1000	0.72	0.74	0.85	0.87	0.93	0.96
100	0.78	0.83	0.78	0.81	0.94	0.94
10	0.67	0.77	0.58	0.68	0.80	0.84
1	0.30	0.52	0.61	0.66	0.80	0.94
0	0.00	0.34	0.52	0.66	0.81	0.92

E.

H2228

Paclitaxel (nM) \ Crizotinib (nM)	0	1	10	100	1000	10000
10000	0.74	0.79	0.82	0.78	0.77	0.87
1000	0.66	0.76	0.76	0.76	0.76	0.77
100	0.69	0.77	0.78	0.78	0.75	0.81
10	0.62	0.71	0.73	0.77	0.74	0.77
1	0.51	0.45	0.62	0.46	0.71	0.76
0	0.00	0.24	0.45	0.45	0.64	0.73

B.

H2228
Ratio Alectinib:Paclitaxel

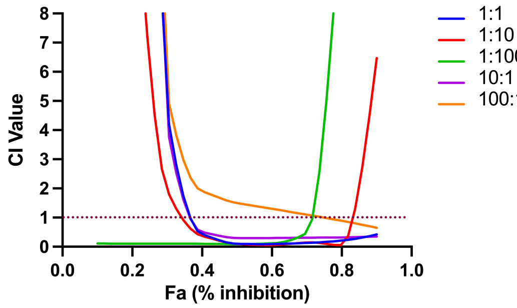

D.

H2228

F.

H2228
Ratio Crizotinib:Paclitaxel

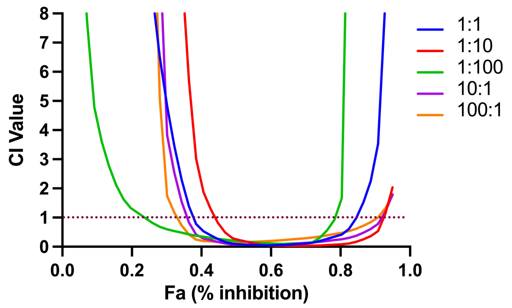

G.

H3122

Paclitaxel (nM) \ Alectinib (nM)	0	1	10	100	1000	10000
10000	0.60	0.71	0.69	0.69	0.84	0.98
1000	0.54	0.62	0.64	0.71	0.76	0.97
100	0.53	0.56	0.63	0.66	0.72	0.97
10	0.43	0.47	0.55	0.60	0.64	0.96
1	0.31	0.44	0.48	0.59	0.66	0.97
0	0.00	0.25	0.32	0.44	0.48	0.96

I.

H3122

Paclitaxel (nM) \ Ceritinib (nM)	0	1	10	100	1000	10000
10000	0.68	0.71	0.77	0.82	0.83	0.97
1000	0.54	0.47	0.46	0.58	0.66	0.83
100	0.43	0.41	0.50	0.50	0.58	0.78
10	0.24	0.30	0.26	0.25	0.53	0.79
1	0.17	0.16	0.29	0.41	0.47	0.77
0	0.00	0.16	0.27	0.32	0.51	0.78

K.

H3122

Paclitaxel (nM) \ Crizotinib (nM)	0	1	10	100	1000	10000
10000	0.56	0.55	0.57	0.62	0.66	0.75
1000	0.51	0.52	0.56	0.61	0.63	0.73
100	0.46	0.48	0.52	0.53	0.58	0.70
10	0.40	0.31	0.37	0.44	0.50	0.66
1	0.32	0.30	0.32	0.39	0.43	0.59
0	0.00	0.05	0.27	0.34	0.40	0.56

H.

H3122
Ratio Alectinib:Paclitaxel

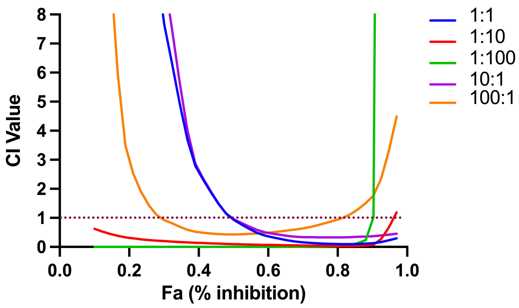

J.

H3122
Ratio Ceritinib:Paclitaxel

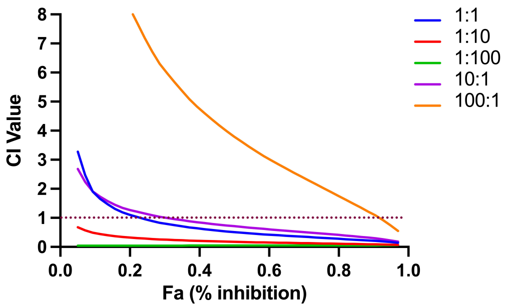

L.

H3122
Ratio Crizotinib:Paclitaxel

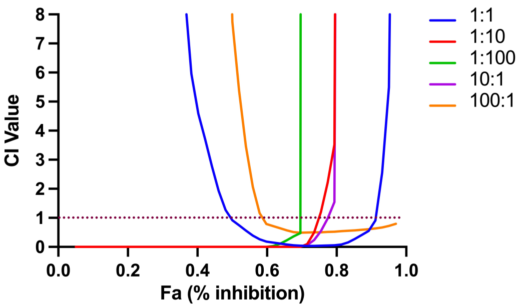

Supplementary Figure S8. Fa and CI values for combination treatment of H2228 and H3122 cells with paclitaxel and ALK inhibitors

A-F. Cell viability data for H2228 cells presented as Fa (% inhibition) in a dose response matrix as determined by Alamar Blue assay for paclitaxel with alectinib (A, B), ceretinib (C, D) or crizotinib (E, F). CI values were calculated using CompuSyn for the indicated ratios of drug combinations and presented in B, D and F as CI:Fa graphs. **G-L**. As for A-F, but for H3122 cells. Data are representative of 3 independent experiments.
